# A Density Functional Benchmark for Dehydrogenation and Dehalogenation Reactions on Coinage Metal Surfaces

**DOI:** 10.1002/cphc.202400865

**Published:** 2024-11-13

**Authors:** Lin Chen, Johanna Rosen, Jonas Björk

**Affiliations:** ^1^ Materials Design Division Department of Physics, Chemistry and Biology IFM Linköping University 58183 Linköping Sweden

**Keywords:** vdWs interactions, DFT, On-surface synthesis, Dehydrogenation, Dehalogenation

## Abstract

The on‐surface synthesis of low‐dimensional organic nanostructures has been extensively investigated through both experimental and theoretical methods, particularly by density functional theory (DFT). However, the complex mixture of interactions often poses challenges within the DFT framework, and there is a knowledge‐gap regarding how the choice of DFT approach affects the computed results. Here, five different approaches including vdW interactions, *i. e*., PBE+D3, PBE+vdW^surf^, rev‐vdWDF2, r^2^SCAN+rVV10 and BEEF‐vdW, are employed to describe three prototypical on‐surface reactions; dehydrogenation of benzene, debromination of bromobenzene, and deiodination of iodobenzene on the (111) facets of the coinage metals. Overall, rev‐vdW‐DF2 outperforms the other methods in describing benzene adsorption, whereas BEEF‐vdW falls short. For dehydrogenation and debromination on Cu(111), all functionals except BEEF‐vdW give reasonable activation energies compared to experiments. A similar trend is observed for Ag(111) and Au(111), with BEEF‐vdW yielding significantly higher activation and reaction energies. For dehalogenation, all the five vdW approaches correctly capture the reactivity trend – Cu(111)*>*Ag(111)*>*Au(111) – and the expected hierarchy between bromobenzene desorption and carbon‐bromine activation. Only BEEF‐vdW fails to predict the faster kinetics of deiodination than the iodobenzene desorption. Our work forms a basis for evaluating density functionals in describing chemical reactions on surfaces.

## Graphical abstract

The complex mixture of interactions involved in on‐surface synthesis poses challenges within the framework of density functional theory. This study evaluates five different approaches, incorporating two types of functionals that account for vdW interactions, to describe three prototypical on‐surface reactions.

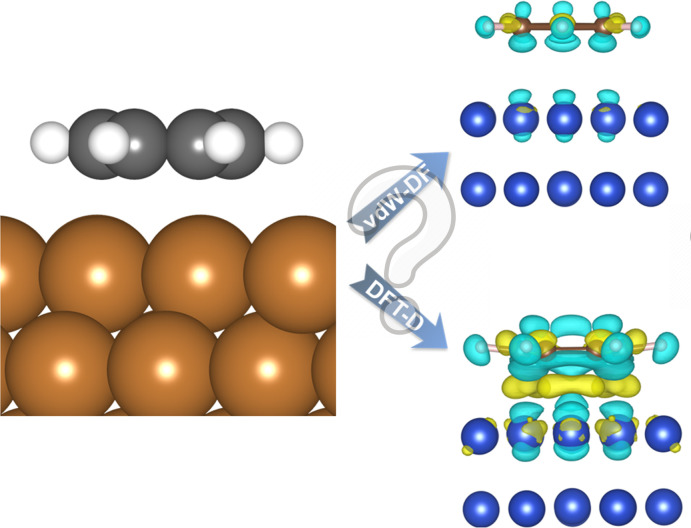

## Introduction

The rapid development of on‐surface synthesis during the past decade has resulted in an increased attention for low‐dimensional covalent organic nanostructures due to their potential utilization in emerging applications within (opto)electronics,^[1]^ semiconductor^[2]^ and spintronic devices,^[3]^ as well as high‐efficient catalysts by designing novel 2D metal‐organic frameworks.^[4]^ Within on‐surface synthesis, normally coinage metal surfaces work as two‐dimensional templates on which the organic molecular precursors undergo thermally activated reactions to form extended organic nanostructures or novel molecules.[^5^, ^6^, ^7^] Unlike strategies for synthesizing low‐dimensional carbon nanomaterials in solution or gas phase, on‐surface synthesis provides a rational bottom‐up approach toward the controllable reactions at the atomic scale with additional characterization possibilities provided by surface science techniques.[^8^, ^9^]

The efficient control of the reactions during on‐surface synthesis requires a thorough understanding of the underlying reaction mechanisms. Combining scanning probe microscopy techniques, particularly scanning tunneling microscopy (STM) and non‐contact atomic force microscopy (nc‐AFM) with spectroscopic techniques and complementary theoretical modeling has made it possible to retrieve detailed information about reactants, products, and, sometimes, intermediates.[^10^, ^11^] However, the short‐lived nature of transition states and many intermediate structures, make them unfeasible to be studied experimentally.^[12]^ In the past decades, theoretical studies, especially first principles calculations within the framework of density functional theory (DFT) have been successfully employed to determine the adsorption configurations of reactant species as well as the thermodynamics and kinetics of on‐surface reactions.[^11^, ^13^, ^14^, ^15^, ^16^, ^17^]

The systems involved in on‐surface synthesis generally includes multiple types of interactions. These are often characterized by complex molecular structures held together by long‐range van der Waals (vdW) interactions, hydrogen bonds and covalent or noncovalent interactions with metal surfaces under ultrahigh vacuum.[^18^, ^19^] Reaching so‐called chemical accuracy is intractable from conventional semi‐local DFT, due to the inability of generalized gradient approximation (GGA) functionals to account for vdW interactions. The vdW interactions originates from the interaction between fluctuating dipoles, which is induced by the instantaneous electron correlations. In some situations, the hybridization of the electronic densities between the organic molecules and transition metal surfaces is weak and vdW interactions are needed even to establish adsorption, which challenges the accuracy of reaction thermodynamics and kinetics calculated by GGA‐level functionals.^[20]^

There are two schools of thought on how to include vdW interactions in standard DFT calculations. The first is referred to as dispersion‐corrected DFT (DFT+D) and treats the problem by adding a correction term including atom‐pairwise dispersion potential of the form *C*_6_ ⋅ *R* ^*−*6^, where *C*_6_ denotes the dispersion coefficient for a pair of atoms and *R* is the inter‐atomic distance.[^21^, ^22^] A damping function must be included to avoid the near‐singularities at small distances. Notably, it has been shown that at binding distances, calculated energies are more sensitive to the parameters in the damping function than the actual *C*_6_ coefficients.^[23]^ In an early version by Grimme, often denoted as DFT+D2,^[21]^ the atomic *C*_6_ coefficients and cutoff radii are obtained in an empirical fashion and do not contain any dependence on the chemical environment, which limits the transferability of the method. In the improved DFT+D3 approach,^[24]^ both the atom pairwise coefficients and cutoff radii are computed from first principles. Particularly, the atom pairwise *C*_6_ coefficients are geometry‐dependent as they are evaluated on the basis of specific coordination numbers around each atom. DFT+D3 has been extensively used thanks to its well‐performed chemical accuracy and low computational cost.^[25]^ Another method (called DFT+vdW) taking the chemical environment into account is that of Tkatchenko and Scheffler,^[22]^ which employs Hirshfeld partitioning of the electron density and calculates the *C*_6_ coefficients for the atomic pair on the fly from the electron density around each atom. To specially target the modeling of adsorbates on surfaces, an extended pairwise vdW approach (called DFT+vdW^surf^) has been proposed by combining the DFT+vdW scheme with the inclusion of nonlocal collective response from the substrate metal surface within the vdW energy tail,[^26^, ^27^] performing effectively beyond the pairwise description.

The second school of thought for incorporating vdW interactions involves introducing a truly non‐local density functional. The so‐called vdW density functional (vdW‐DF) is based solely on the electron density of the system.^[28]^ In its original form (vdW‐DF1) proposed by Dion *et al*.,^[28]^ the exchange‐correlation (*xc*) energy comprises revPBE exchange, and the local‐density approximation (LDA) for local correlation and a fully non‐local functional accounting for all non‐local correlation. The seamless connection between long‐range and short‐range interactions eliminates the need for a damping function. The vdW interactions in this framework are described by a nonlocal correlations term, which is accounted within the exchange‐correlation energy in a self‐consistent manner.^[29]^ The self‐consistent potential derived from vdW‐DF allows for efficient evaluation of forces and the stress tensor, incorporating vdW interactions.^[29]^ Following its original version vdW‐DF1, several improvements have been proposed, primarily related to the choice of semi‐local exchange part and modifications of the vdW kernel (governing the non‐local correlation part). For example, optB86b‐vdW,^[30]^ optB88‐vdW^[31]^ and vdW‐DF‐cx^[32]^ combine the original vdW kernel with different semi‐local exchange functionals. vdW‐DF2^[33]^ employes a reoptimized parameter in the vdW kernel alongside PW86R exchange, while rev‐vdW‐DF2^[34]^ is a revised version that uses an exchange resembling that optB86b together with the kernel of vdW‐DF2. The rVV10 method^[35]^ combines PW86R exchange and nonlocal correlation with a kernel of different analytical form. The nonlocal correlation of rVV10 has also been combined with the SCAN meta‐GGA functionals, such that r^2^SCAN+rVV10.^[36]^

Among various coupling reactions within on‐surface synthesis, Ullmann‐type coupling reactions have been recognized as the most frequently employed with high efficiency and robustness, which involves dehalogenation and subsequent C−C bond formation.^[37]^ An archetypical example is the on‐surface synthesis of graphene nanoribbons, which are generally obtained by on‐surface Ullmann coupling followed by cyclodehydrogenation.^[38]^ A nanographene has also been obtained via on‐surface cyclodehydrogenation of a prototypical polyphenylene.^[5]^ Dehydrogenation reactions are also essential in many other on‐surface synthesis protocols, such as formation of higher order acenes.^[39]^ Therefore, accurately describing both dehalogenation and dehydrogenation reactions from DFT calculations is essential within the field of on‐surface synthesis.^[40]^ However, the diverse inorganic and organic components at the interfaces make the choice of vdW approaches as well as *xc* functionals challenging. How calculated results depend on the treatment of vdW interactions and the choice of *xc* functionals is crucial for the reliability of theoretical studies. In fact, the choices of computational methods that work successfully for one of the components often yield poor results for the other,^[16]^ which calls for a comparative study on the performance of various vdW‐inclusive density functionals in the field of on‐surface synthesis.

In this work, we investigate how five different vdW approaches describe dehydrogenation and dehalogenation, more specifically, dehydrogenation of benzene as well as dehalogenation of bromobenzene and iodobenzene, on three metal substrates (Cu, Ag and Au). We selected a diverse set of vdW‐inclusive density functionals, comprising two semiclassical *C*_6_ approaches (PBE+D3^[24]^ and PBE+vdW^surf[26,27]^), as well as three nonlocal‐density‐based methods (rev‐vdW‐DF2,^[34]^ r^2^SCAN+rVV10^[36]^ and BEEF‐vdW^[41]^). In the class of dispersion‐corrected DFT methods, PBE+vdW^surf^ was selected since it was developed with adsorption on metal surfaces in mind.[^26^, ^27^] PBE+D3 was selected for its simplicity and widespread use, providing reliable results and being one of the most commonly applied vdW methods today.^[16]^ Among non‐local density functionals, rev‐vdW‐DF2 was selected since it has shown superior performance, compared to other non‐local functionals, in both weakly and strongly bound systems.^[42]^ Compared with vdW‐DF family of nonlocal correlation, the rVV10 methods are more flexible and simple in form by optimizing two fixed parameters with reference systems.^[35]^ The r^2^SCAN+rVV10 method, a combination of the second‐revised SCAN meta‐GGA, with the flexible rVV10 vdW method, was included for its demonstrated accuracy across diverse bonding environments and its numerical stability – advantageous over SCAN and rSCAN.^[36]^ To our knowledge, this is the first application of r^2^SCAN+rVV10 to study dehydrogenation and dehalogenation in this context. Lastly, BEEF‐vdW,^[41]^ which optimizes the semiempirical exchange correlation functional using training data sets from machine learning methods, was selected due to its widespread use in hydrogenation and dehydrogenation reactions for various systems.^[43]^ Our results show that rev‐vdW‐DF2 most accurately describes the adsorption of benzene, while BEEF‐vdW performs worst. For the bromobenzene and iodobenzene adsorption on Ag(111) and Au(111), all functionals except BEEF‐vdW give roughly similar adsorption geometries. For dehydrogenation on Cu(111), all functionals except BEEF‐vdW give a reasonable prediction of the activation energy compared to experimental estimations. A similar trend is observed for Ag(111) and Au(111), with BEEF‐vdW yielding significantly more positive activation and reaction energies. For dehalogenation reactions, all of the five vdW approaches correctly capture the reactivity trend of the three metal surfaces: Cu(111) is the most reactive, followed by Ag(111) and Au(111) is the least reactive.

## Computational Methods

Density functional theory calculations were performed with the Vienna Ab‐initio Simulation Package (VASP).^[44]^ The Kohn‐Sham orbitals were expanded with plane waves using a 450 eV energy cut‐off and the interaction between the valence electrons and the cores was described with the plane augmented wave (PAW) approach.[^45^, ^46^] The number of valence electrons considered in the calculations are 1 (H), 4 (C), 11 (Cu), 7 (Br), 11 (Ag), 7 (I) and 11 (Au).

The Cu(111), Ag(111) and Au(111) surfaces were built using the calculated bulk lattice constants, which are summarized for the five density functionals in Figure S1. Compared with experimental lattice constants, the closest value was obtained by rev‐vdW‐DF2 for Cu and Ag, and by PBE+D3 for Au. BEEF‐vdW overestimates the lattice constants for all three metals. Cu(111), Ag(111) and Au(111) surfaces were modeled by periodically repeated *p*(5×5) surface cells with a four‐layer slab separated by a 15 Å vacuum layer. The atoms in the bottom two layers were kept fixed during the geometry optimization. The Brillouin zone was sampled using the Monkhorst–Pack^[47]^ scheme with a 3×3×1 *k*‐point for all considered surface cells. Structures were optimized with the conjugate gradient method and geometries were considered to be converged when the largest force on the atoms in the calculated system is smaller than 0.02 eV/Å. The electronic self‐consistency loop stops when the total energy change between two subsequent steps is smaller than 1×10^−5^ eV. We also performed an additional static calculation with stricter convergence criterion 1×10^−7^ eV and the energy change is within 1×10^−4^ eV. Reaction barriers were calculated by a combination of the climbing image nudged elastic band and the Dimer method as implemented in the transition‐state tools of VASP.[^48^, ^49^] The climbing nudged elastic band calculations were completed with a convergence criterion of 1×10^−5^, while the convergence criterion for the Dimer calculations was set to a stricter value of 1×10^−7^ eV. Harmonic vibrational frequencies were computed using the finite‐difference approach. The surface atoms were fixed during the vibrational analysis. The transition states were confirmed by vibrational analysis showing one imaginary frequency along the reaction coordinate.

## Results and Discussions

### Adsorption of Benzene on Cu(111), Ag(111) and Au(111)

As the simplest aromatic hydrocarbon, benzene is an ideal probe model for aromatic molecules. The adsorption of benzene on metal surfaces is therefore prototypical for the interactions between functional aromatic compounds and metal surfaces. On the (111) facets of coinage metals, it is generally concluded that benzene adsorbs with the aromatic ring parallel to the surface, which suggests that the out‐of‐plane benzene pi orbitals are mainly involved in the bonding process.^[50]^ The parallel adsorption geometry suggests that benzene has an in‐plane rotational degree of freedom at low coverage.^[51]^ To conclude what is the most stable adsorption configuration, we considered benzene centered above a top, bridge, HCP hollow, and FCC hollow site of respective surface, as well as two rotational configurations. In total, eight high‐symmetry adsorption configurations were considered (see Figure S2), denoted as BR (bridge), BR_−_R, FCC, FCC_−_R, HCP, HCP_−_R, TOP and TOP_−_R. The suffix with (without)“_−_R” denotes 30 (or 0)‐degree rotation of benzene with respect to the *<*110*>* direction.

The adsorption energies of benzene in the eight high‐symmetry configurations on Cu(111), Ag(111) and Au(111) surfaces from five different vdW functionals are shown in Figure S3, S4 and S5, respectively. The adsorption energies for the eight sites vary within 0.2 eV on the three metal surfaces and generally the HCP site has the lowest adsorption energy from the considered vdW functionals. However, by comparing the most favorable site for benzene adsorption from literature with our work it is not possible to reach a consensus. Berland et al.^[51]^ found FCC and HCP hollow from vdW‐DF are preferred for benzene adsorption on Cu(111) while Carter et al.^[52]^ showed FCC_−_R from vdW‐DF and TOP from vdW‐DF2 are the most favorable sites on Cu(111), respectively. Miller et al.’s DFT+D2 and optB88‐vdWDF calculations showed HCP_−_R is most stable site for benzene adsorbed on Ag(111).^[53]^ By using PBE+vdW^surf^, Carrasco et al.^[54]^ predicted BR_−_R site to be the most favorable site for benzene adsorption on Cu(111), Ag(111) and Au(111).

One of the reasons for the discrepancy on the site preference may be due to the different choices of the vdW functional. It is known that benzene adsorbs physically on (111) coinage metal surfaces, which indicates the dispersion interaction is an indispensable part among multiple types of interactions in the system.^[54]^ Different approaches accounting for vdW interactions give distinct descriptions of the dispersion interaction between benzene and the metal substrates, which yields an inconsistency of the most preferable adsorption site. Another important aspect is the flat potential energy surface (PES) around the equilibrium adsorption height (see Figure S6) due to the weak benzene‐metal interaction, which makes it challenging to relax structures into energy minima even for strict convergence criteria. This is particularly important for finding the correct adsorption height for which it is necessary to map the PES.

In Figure S6, the PES for benzene adsorbed on (111) coinage metal surfaces at HCP site as a function of the benzene height from rev‐vdW‐DF2 are shown. The curves clearly show the physisorption character of benzene adsorption on these three metal surfaces. The lowest‐energy adsorption heights for benzene adsorbed on Cu(111), Ag(111) and Au(111) from rev‐vdW‐DF2 are 2.8 Å, 3.0 Å and 2.9 Å, respectively. The PES around the lowest‐energy adsorption height on Cu(111), Ag(111) and Au(111) from all five different vdW approaches are shown in Figure S7, S8 and S9, respectively. By probing the PES as a function of the adsorption height, the equilibrium adsorption geometry can be obtained.

**Figure 1:**
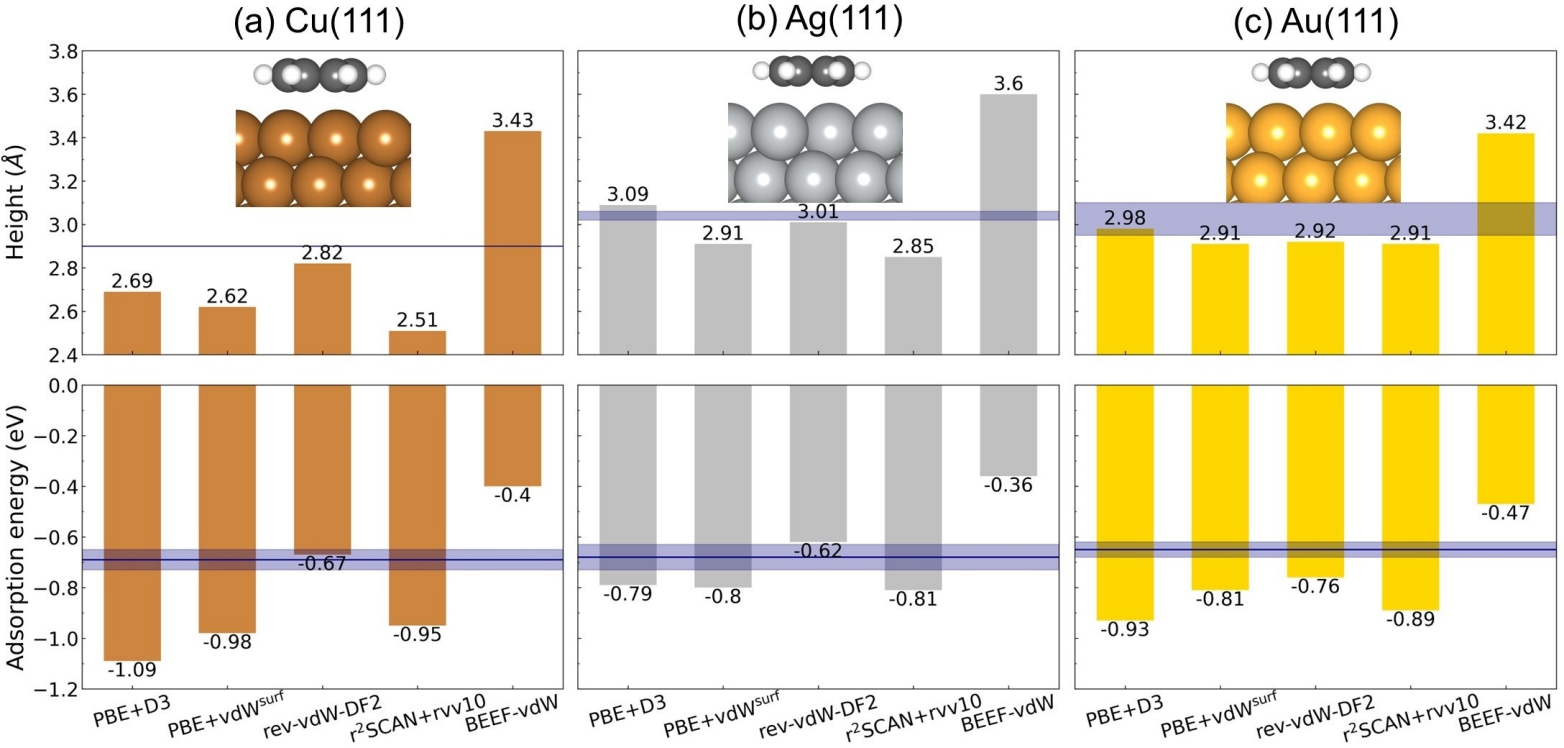
The equilibrium height (upper panel) and adsorption energy (lower panel) of benzene adsorbed on (a) Cu(111), (b) Ag(111) and (c) Au(111) at hcp hollow site from different density functionals. The height of benzene is determined by calculating the averaged carbon‐metal(top layer metal atoms) distance. The horizontal bands indicate the experimental binding heights and adsorption energies. The experimental adsorption energies are taken from recent interpretation of temperature programmed desorption (TPD) spectra in the limit of vanishing surface coverage with statistical error.^[50]^ The experimental heights are also shown for comparison: Cu,^[55]^ Ag^[50]^ and Au.^[56]^ It should be noted that the adsorption height for Ag is directly measured from normal‐incidence x‐ray standing wave but the values for Cu and Au are extrapolated from experimental change in work function values combined with vdW‐DF calculations. The figures in upper panel and in lower panel share the same range of y axis, respectively. Atom color codes: Au (gold), Ag (silver), Cu (peru), C (dimgray) and H(white).

The equilibrium adsorption height and adsorption energies from different vdW approaches for the three metal surfaces are shown in Figure 1. Experimental values are also indicated for comparison. The experimental heights for benzene adsorbed on Cu(111), Ag(111) and Au(111) are 2.9 Å,^[55]^ 3.02–3.06 Å^[50]^ and 2.95–3.10 Å,^[56]^ respectively. It should be noted that the adsorption height for Ag is directly measured from near‐incidence x‐ray wave adsorption but the values for Cu and Au are extrapolated from experimental change in work function values combined with vdW‐DF calculations. The experimentally estimated adsorption energies, from temperature‐programmed desorption, are −0.69±0.04 eV for Cu(111), −0.68±0.05 eV for Ag(111) and −0.65±0.03 eV for Au(111).^[50]^ At equilibrium adsorption heights, the binding strength of benzene on the three metals is determined by a balance between repulsive and attractive forces. The slightest difference of the adsorption height for the three metals is possibly originated from the different vdW radii of the metal atoms. Compared to the experimental values, rev‐vdW‐DF2 gives better descriptions of both adsorption height and adsorption energies on Cu(111) than the other four vdW approaches. For Ag(111), PBE+D3 and rev‐vdW‐DF2 give proper predictions on the height and rev‐vdW‐DF2 produces the closest adsorption energy with respect to the experimental results. For Au(111), all vdW functionals except BEEF‐vdW provide reasonable adsorption heights but none of the functionals give accurate adsorption energies, although rev‐vdW‐DF2 gives the value closest to the experiments. Although experimental adsorption energies includes the contribution from zero‐point energy and thermal energy, the contribution is rather small for this type of physisorption, justifying the direct comparison. We note that none of these five vdW approaches captures the correct trend (Cu(111)*>*Ag(111)*>*Au(111)) in binding strength of benzene on the three metal surfaces. For example, rev‐vdW‐DF2 predicts the trend as Au(111)*>*Cu(111)*>*Ag(111) and PBE+D3 predicts the trend as Cu(111)*>*Au(111)*>*Ag(111). It can also be concluded that dispersion‐corrected DFT methods predict the strongest adsorption on Cu(111) but vdW‐inclusive nonlocal density functionals predict the strongest adsorption on Au(111) except r^2^SCAN+rVV10. This is in good agreement with the other theoretical work,^[54]^ which also shows the strongest adsorption of benzene on Au(111) by nonlocal density functionals (including optPBE‐vdW, optB88‐vdW and optB86b‐vdW) and the strongest adsorption on Cu(111) by dispersion‐corrected DFT (including PBE+vdW and PBE+vdW^surf^). Overall, rev‐vdW‐DF2 shows a reasonable performance regarding both adsorption geometries and energies. It should be noted that BEEF‐vdW overestimates the adsorption height and underestimates the adsorption energies significantly, which indicates that BEEF‐vdW performs poorly for these systems.

### Adsorption of Bromobenzene and Iodobenzene on Cu(111), Ag(111) and Au(111)

**Figure 2:**
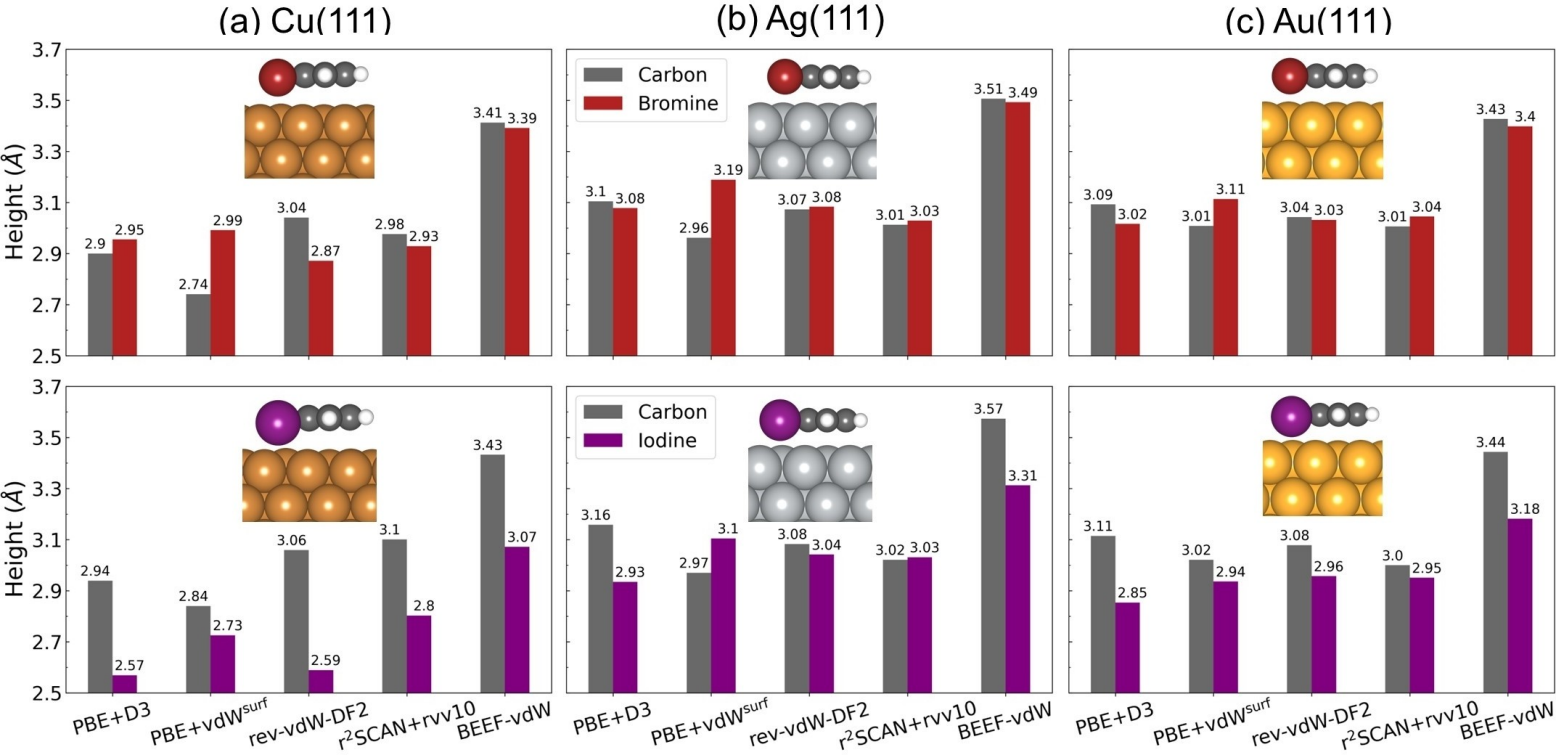
The height of bromobenzene (upper panel) and iodobenzene (lower panel) adsorbed at hcp hollow site on (a) Cu(111), (b) Ag(111) and (c) Au(111). The individual height of the bromine atom (red bar), the iodine atom (purple bar) and the average height of carbon atoms (grey bar) from different density functionals are shown for each system, respectively. The adsorption configurations from rev‐vdW‐DF2 are shown in the inset. The figures in upper panel and in lower panel share the same range of y axis, respectively. Atom color codes: Au (gold), I (purple), Ag (silver), Br (brown), Cu (peru), C (dimgray) and H(white).

**Figure 3:**
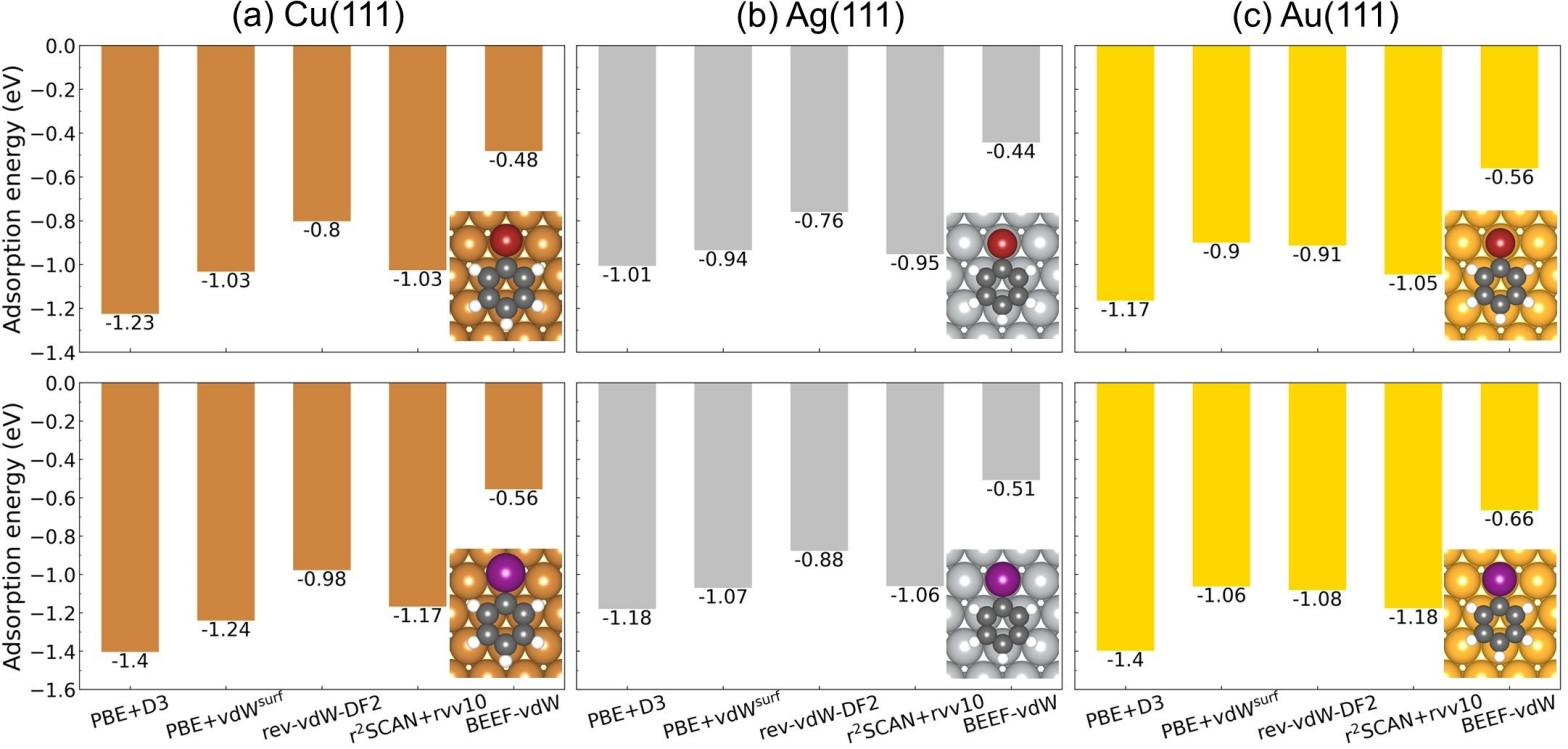
The adsorption energy of bromobenzene (upper panel) and iodobenzene (lower panel) at hcp hollow site on (a) Cu(111), (b) Ag(111) and (c) Au(111) from different density functionals. The adsorption configurations from rev‐vdW‐DF2 are shown in the inset. The figures in upper panel and in lower panel share the same range of y axis, respectively. Atom color codes: Au (gold), I (purple), Ag (silver), Br (brown), Cu (peru), C (dimgray) and H(white).

It has been shown that aryl halides preferably adsorb with their halogen atoms at top sites of (111) coinage metal surfaces.[^15^, ^57^] Therefore, the adsorption geometries of bromobenzene and iodobenzene on (111) coinage metal surfaces are obtained by optimizing the equilibrium structures of benzene adsorption on hcp site of these metal surfaces with one of the hydrogen atom ontop the metal atom replaced by a halogen atom. The individual height of the halogen atom and the average height of carbon atoms with respect to the metal surface from different vdW approaches are shown in Figure 2 and the corresponding adsorption energies in Figure 3. The height of Br slightly deviate from the average height of carbon atoms for both bromobenzene on Cu(111) from all functionals except PBE+vdW^surf^, indicating that bromobenzene adsorbs nearly parallel to the surface. It has also been experimentally observed that bromobenzene adsorb at low coverages with its plane parallel to the Cu(111) surface,^[58]^ which implies that PBE+vdW^surf^ fails to give a correct description. For the iodobenzene adsorption on Cu(111), the height of I is significantly lower than the average height of carbon atoms for all five vdW functionals, resulting in the tilted adsorption geometries of iodobenzene on Cu(111). Compared to Cu(111), bromobenzene adsorbs with much flatter geometries on Ag(111) surface and Au(111) surface from all functionals except PBE+vdW^surf^, which is indicated by the similar heights of Br and C atoms. In general, for the bromobenzene and iodobenzene adsorption on Ag(111) and Au(111), all functionals except BEEF‐vdW give roughly similar adsorption heights.

Figure 3 shows that I interacts more strongly with the coinage metal than Br, as indicated by their adsorption energies across all vdW approaches. The stronger interaction results in the lower height of I than Br (Figure 2). PBE+D3 predicts the lowest adsorption energies, which is consistent with the description of benzene adsorption in Figure 1. BEEF‐vdW predicts the highest adsorption energies for all cases, which explains the larger height for Br and I than other vdW approaches.

To understand the different adsorption geometries from the five vdW functionals, projected electronic density of states for pure metal surfaces and adsorbed surfaces are shown in Figure S10–S21 and the d band center is also annotated in each figure. It has been widely acknowledged d‐band center theory has improved our understanding on the interactions between the adsorbates and transition metals.^[59]^ The d‐band center shift is calculated by the adsorbates‐induced d‐band center changes on (111) coinage metal surface and the d‐band center shift with respect to the height of adsorbed molecule exhibits a linear relationship (see Figure S22) for each system. It can be concluded that the larger d‐band center shift is strongly related to the lower adsorption height and vice versa. Notably, the nonlocal density based vdW approaches follows the linear trend better than the dispersion corrected vdW methods (specially for bromobenzene on Au(111)), indicating a possible correlation with the self‐consistency of the electronic structures from the nonlocal density functional.

### Dehydrogenation of Benzene on Cu(111), Ag(111) and Au(111)

**Figure 4:**
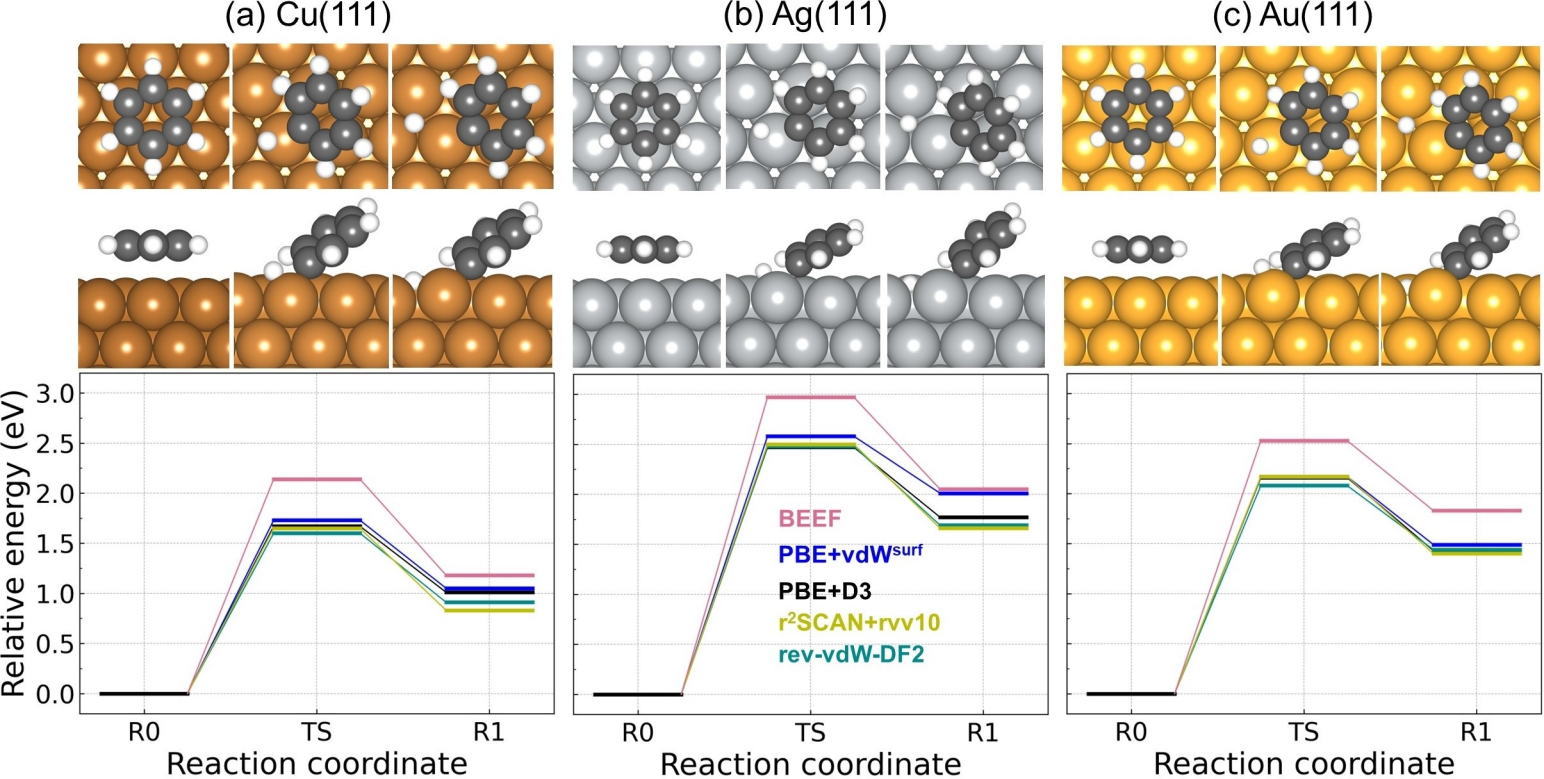
The potential energy landscape for dehydrogenation reaction of benzene on (a) Cu(111), (b) Ag(111) and (c) Au(111). The configurations from rev‐vdW‐DF2 are shown above each potential energy landscape. The top and side view of each structure are shown in upper and lower panel, respectively. The three potential energy landscapes share the same range of y axis. Atom color codes: Au (gold), Ag (silver), Cu (peru), C (dimgray) and H(white).

Experimentally, thermal activation of the C−H bond scission is not possible to be captured on these three surfaces as benzene desorbs before being dehydrogenated.^[60]^ Although with the use of STM the tunneling‐current‐induced dehydrogenation has been observed by the electron dosing into the molecule,[^61^, ^62^, ^63^] it is of interests to have a quantitative knowledge on the activation energy and the reaction energy of the dehydrogenation. Here, the dehydrogenation of benzene is theoretically investigated by performing the first hydrogen abstraction, which leads to a phenyl radical C_6_H_5_ and a hydrogen atom adsorbed on the surface. The dehydrogenation reaction energy landscapes on (111) coinage metal surfaces from five vdW approaches are shown in Figure 4. The activation energies and reaction energies are summarized in Table 1 as well. On Cu(111) (Figure a), all approaches except BEEF‐vdW give a similar reaction landscape with the activation energy within 1.60–1.75 eV and the reaction energy within 0.80–1.05 eV, while BEEF‐vdW gives 2.15 eV and 1.20 eV for the activation and reaction energy, respectively. The transition‐state (TS) geometry shows C and H atoms share almost the same Cu on top adsorption site, which indicates a stretch mode of the loose C−H bond. The tilted adsorption configuration of phenyl results from a combination of the *σ* bonding between the phenyl radical with the surface and the *π*‐bonding contribution from the ring.^[64]^ Without any vdW corrections, the activation energy for dehydrogenation of benzene on Cu(111) has previously been calculated to be 2.2 eV,^[65]^ which is similar with the value from BEEF‐vdW. However, it has been concluded from the experimental observations that the cyclodehydrogenation of a prototypical cyclic polyphenylene on Cu(111) proceeds with barriers much less than 2.2 eV.^[5]^ Although a direct comparison between the cyclic polyphenylene and benzene is not possible, it give a strong indication that BEEF‐vdW fails to describe C−H bond scission of benzene on Cu(111). The failure of BEEF‐vdW for this reaction could originate from its dramatic overestimation of the adsorption height of benzene on Cu(111) as it requires extra amount of energy to move benzene close to the surface from its equilibrium adsorption height (see Figure S7) compared with other four vdW approaches. Therefore, the accuracy of the calculated activation energy for dehydrogenation of benzene on Cu(111) directly depends on a precise prediction of the adsorption geometry, underscoring the necessity of a dependable vdW functional that describes the adsorption correctly. The barrier of 1.60–1.75 eV for dehydrogenation of benzene on Cu(111) is within in a more reasonable range of the experimental estimations.^[5]^

**Table 1:** Calculated activation energies (E_a_) and reaction energies (▵E) for dehydrogenation, debromination and deiodination on Cu(111), Ag(111) and Au(111), obtained with different vdW approaches, as indicated.

Dehydrogenation	Cu(111)		Ag(111)		Au(111)	
	E_a_(eV)	▵E(eV)	E_a_(eV)	▵E(eV)	E_a_(eV)	▵E(eV)
PBE+D3	1.67	1.01	2.47	1.77	2.16	1.43
PBE+vdW^surf^	1.73	1.05	2.58	2.01	2.17	1.49
rev‐vdW‐DF2	1.60	0.91	2.48	1.69	2.08	1.44
r^2^SCAN+rVV10	1.65	0.83	2.50	1.66	2.17	1.40
BEEF‐vdW	2.14	1.18	2.97	2.05	2.53	1.83

Debromination	Cu(111)		Ag(111)		Au(111)	
	E_a_(eV)	▵E(eV)	E_a_(eV)	▵E(eV)	E_a_(eV)	▵E(eV)
PBE+D3	0.67	−0.81	1.05	−0.20	1.30	0.06
PBE+vdW^surf^	0.70	−0.85	1.14	−0.03	1.28	0.11
rev‐vdW‐DF2	0.61	−0.94	0.94	−0.29	1.13	−0.02
r^2^SCAN+rVV10	0.64	−1.20	1.12	−0.37	1.27	−0.12
BEEF‐vdW	0.89	−0.64	1.14	−0.12	1.36	0.11

Deiodination	Cu(111)		Ag(111)		Au(111)	
	Ea(eV)	▵E(eV)	Ea(eV)	▵E(eV)	Ea(eV)	▵E(eV)
PBE+D3	0.36	−0.96	0.71	−0.39	0.94	−0.25
PBE+vdW^surf^	0.49	−0.96	0.78	−0.23	0.84	−0.21
rev‐vdW‐DF2	0.36	−1.03	0.63	−0.46	0.75	−0.32
r^2^SCAN+rVV10	0.38	−1.30	0.72	−0.64	0.82	−0.43
BEEF‐vdW	0.58	−0.80	0.84	−0.29	1.07	−0.21

The calculated reaction landscape for the dehydrogenation of benzene on Ag(111) is shown in Figure 4(b). The reaction is more endothermic, reflected by more positive reaction energies compared to Cu(111) for all five vdW methods. The activation energy from the majority of the functionals (all except BEEF‐vdW) are found within the interval 2.45–2.60 eV, about 1 eV larger than on Cu(111). It should be noted that PBE+vdW^surf^ stands out by predicting a similar reaction energy as BEEF‐vdW but much lower barrier than BEEF‐vdW. Turning to the dehydrogenation of benzene on Au(111) (see Figure 4(c)), the reaction barriers and reaction energies from all vdW functionals except BEEF‐vdW are within the interval 2.05–2.20 eV and 1.40–1.50 eV, respectively. The barrier and reaction energy from BEEF‐vdW is ∼0.5 eV larger than by the other approaches.

**Figure 5:**
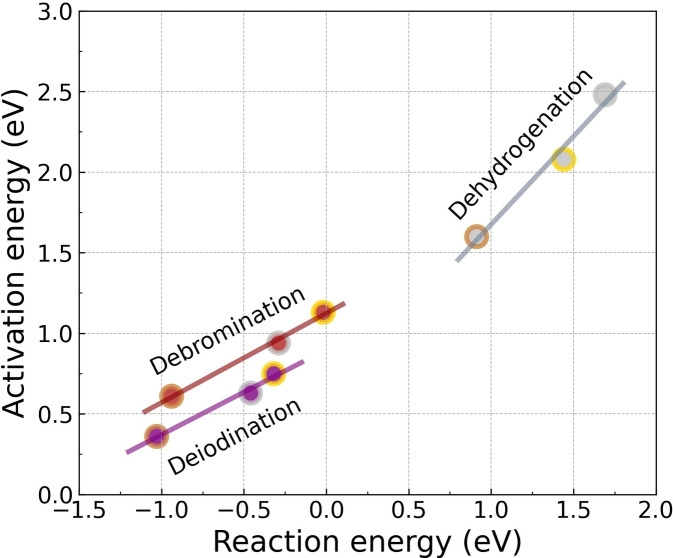
BEP relation for dehydrogenation (silver marker), debromination (brown marker) and deiodination (purple marker) on Cu(111), Ag(111) and Au(111). The peru, silver and gold edge of the markers shows the reaction on Cu(111), Ag(111) and Au(111), respectively. All data are calculated by rev‐vdW‐DF2.

For all methods, Cu(111) has the highest reactivity for the catalyzed dehydrogenation of benzene molecules into chemically active species and Ag(111) has the lowest reactivity. It is also experimentally observed that Cu(111) is more active than Ag(111) and Au(111) for C−H bond cleavage both during cyclodehydrogenation and oligomerization processes,[^66^, ^67^] corroborating our computed results. The activation energy with respect to the reaction energy on these three metal surfaces is found to exhibit a linear relation (see Figure 5 silver line), manifesting the Brønsted‐Evans‐Polanyi (BEP) relation. The BEP relation corroborates that Au(111) is more active than Ag(111) for C−H bond activation, which possibly originates from the stronger interaction of the Au−C bond than the Ag−C bond.^[68]^ The stronger interaction between Au and C stabilizes the transition state and the phenyl species on Au, which reduces the reaction energy and therefore reduces the activation energy. Although the adsorption energies of benzene from PBE+D3,^[24]^ PBE+vdW^surf^, rev‐vdW‐DF2 and r^2^SCAN+rVV10 differ from each other, the similar reaction and activation energies indicate the cancellation among different descriptions of the total energy components by taking the relative energies along the reaction landscape. I.e., the description of the adsorption geometry may be the most crucial factor affecting the calculated reactivity.

### Dehalogenation of Bromobenzene and Iodobenzene on Cu(111), Ag(111) and Au(111)

**Figure 6:**
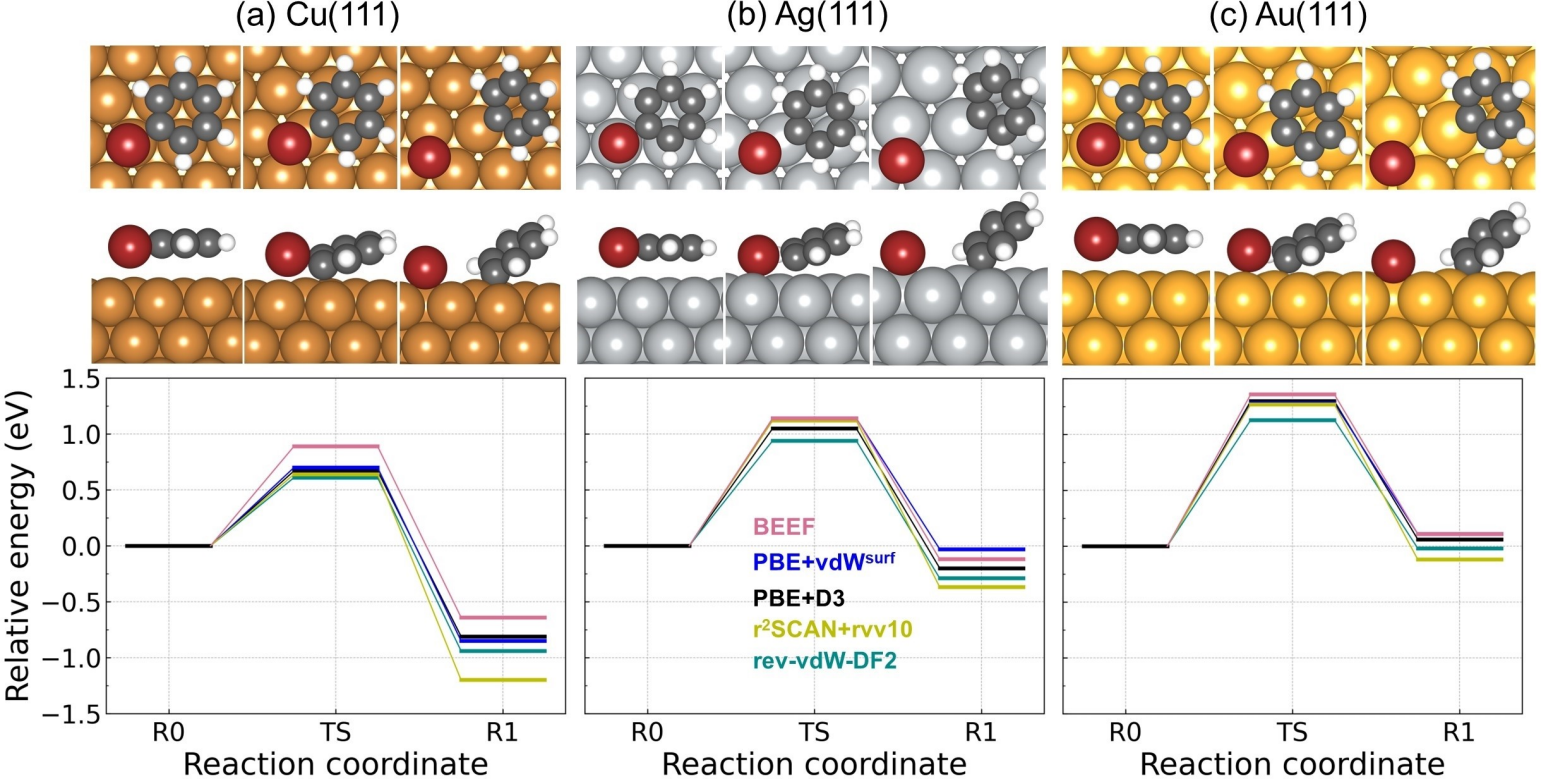
The potential energy landscape for debromination reaction of bromobenzene on (a) Cu(111), (b) Ag(111) and (c) Au(111). The configurations from rev‐vdW‐DF2 are shown above each potential energy landscape. The top and side view of each structure are shown in upper and lower panel, respectively. The three potential energy landscapes share the same range of y axis. The activation energy for C−Br bond scission of 4,4”‐dibromo‐para‐terphenyl on Cu(111) has been estimated to be in the interval 0.5 eV–0.7 eV,^[73]^ which addresses that BEEF‐vdW overestimates the barrier on Cu(111). Atom color codes: Au (gold), Ag (silver), Br (brown), Cu (peru), C (dimgray) and H(white).

**Figure 7:**
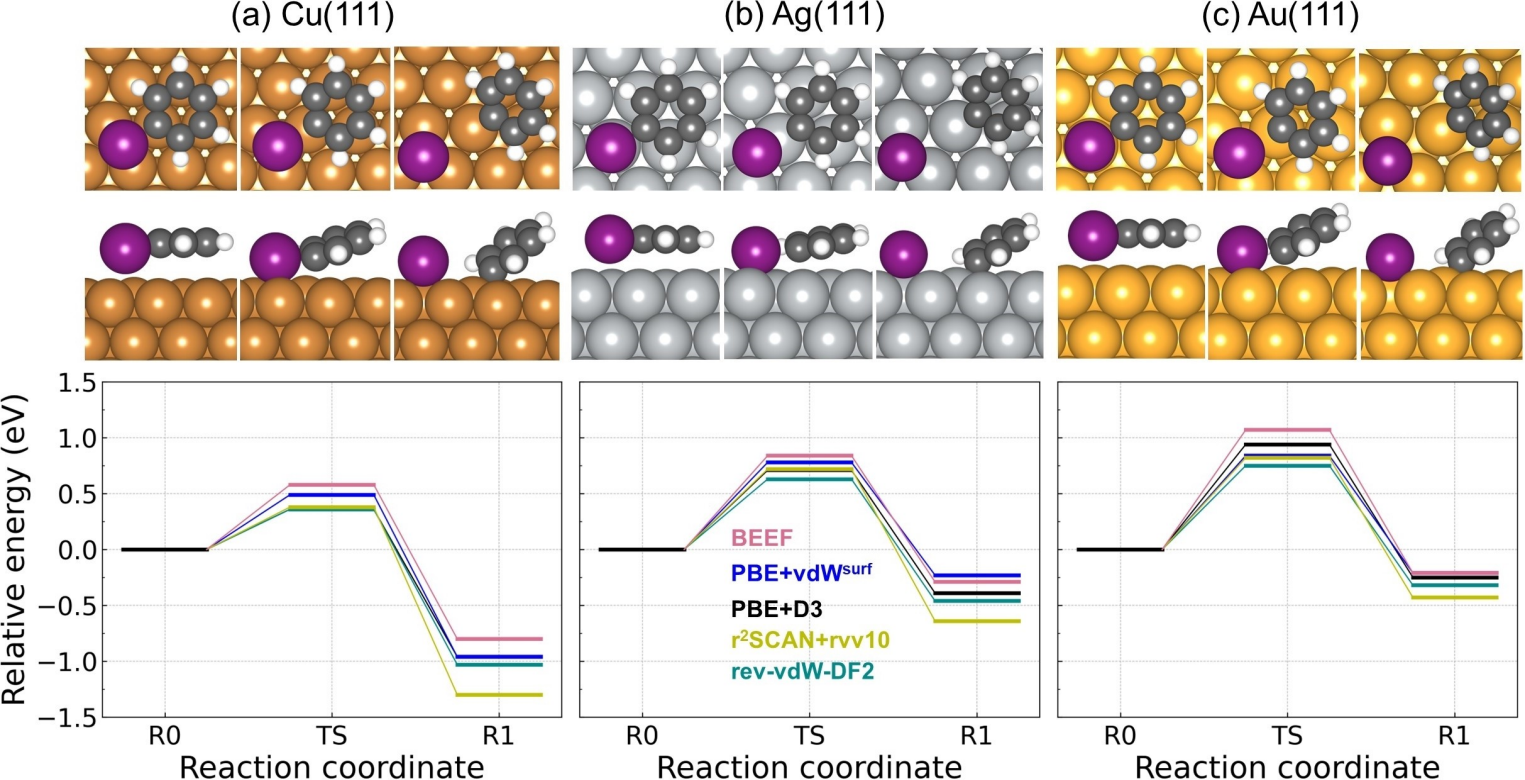
The potential energy landscape for deiodination reaction of iodobenzene on (a) Cu(111), (b) Ag(111) and (c) Au(111). The configurations from rev‐vdW‐DF2 are shown above each potential energy landscape. The top and side view of each structure are shown in upper and lower panel, respectively. The three potential energy landscapes share the same range of y axis. Atom color codes: Au (gold), I (purple), Ag (silver), Cu (peru), C (dimgray) and H(white).

It is generally agreed that the weak carbon‐halogen (Br, I) bond and strong metal‐halogen bond make the carbon‐halogen cleavage more facile than carbon‐hydrogen scission on the coinage metal surfaces.^[69]^ Here, the dehalogenation of bromobenzene (Figure 6) and iodobenzene (Figure 7) are investigated on Cu(111), Ag(111) and Au(111) for the five methods, respectively. The results are summarized in Table 1. The lower activation energies and more exothermic reaction energies for debromination and deiodination on each metal surface demonstrate C−Br and C−I bond breaking are favored over C−H bond breaking both thermodynamically and kinetically.

For debromination reaction on Cu(111), all vdW functionals except BEEF‐vdW give a similar activation energy within 0.60–0.70 eV and BEEF‐vdW gives 0.89 eV. As to the reaction energy, r^2^SCAN+rVV10 gives lowest value by −1.20 eV and BEEF‐vdW gives highest value by −0.64 eV. The other three functionals give a reaction energy between −0.95 and −0.80 eV. On Ag(111) and Au(111), the activation energy and the reaction energy from BEEF‐vdW slightly deviate from the results of the others. The activation energies on Ag(111) for all five functionals range from 0.95–1.15 eV and the reaction energies range from −0.40–0 eV. On Au(111), the activation energies are within the interval 1.10–1.40 eV and the reaction energies are between −0.10 and 0.10 eV. Overall, rev‐vdW‐DF2 give the lowest debromination activation energy for all three metal surfaces and r^2^SCAN+rVV10 gives lowest reaction energy for all surfaces. It can be concluded that all of the five vdW approaches give the same prediction on the debromination reactivity trend among the three catalysts, which is Cu(111)*>*Ag(111)*>*Au(111). As desorption of bromobenzene occurs before the debromination on Ag(111) and Au(111), it is not possible to compare the experiments with our theoretical prediction on the reactivity trend for debromination of bromobenzene. It is noteworthy that all functionals correctly describe the fact that the desorption of bromobenzene is more facile than the debromination on Ag(111) and Au(111). Instead, the abstraction of Br from brominated derivatives of polycyclic aromatic hydrocarbons could be used as experimental references. We found that our predicted reactivity trend agrees well with the experimental studies, which demonstrate C−Br bond scission proceeds on Cu(111) at lower temperatures but requires elevated temperatures on Ag(111) and even higher temperatures on Au(111).[^70^, ^71^, ^72^] Notably, the reactivity trend of debromination follows the BEP relation on the three surfaces (see Figure 5 brown line). More specifically, it has been experimentally observed that the scission of the C−Br bonds within brominated derivative of polycyclic aromatic hydrocarbon at low coverages on Cu(111) occurs between 240 K–300 K dependent on various intermediate states,[^70^, ^73^, ^74^, ^75^] however, additional thermal annealing between 285 K–310 K is required on Ag(111)[^76^, ^77^] and at least ∼300 K is required on Au(111).[^75^, ^78^] The activation energy for C−Br bond scission of 4,4”‐dibromo‐para‐terphenyl on Cu(111) has been estimated to be in the interval 0.5 eV–0.7 eV,^[73]^ indicating that BEEF‐vdW overestimates the barrier. Although a quantitative assessment is not possible for Ag(111) and Au(111), it is reassuring that all methods give the correct trend between the three surfaces.

It is well known that the C−I bonds are more easily dissociated than the C−Br bonds both in gas phase and on coinage metal surfaces,[^79^, ^80^] which is corroborated by our calculated deiodination reaction landscape (see Figure 7). On Cu(111), the activation energies for deiodination from all five methods are within the interval 0.35–0.60 eV and the reaction energies are between −1.30 and −0.80 eV. On Ag(111), the activation energies and the reaction energies range from 0.60–0.85 eV and −0.65–−0.25 eV, respectively. On Au(111), the activation energies and the reaction energies are within the range from 0.75–1.10 eV and −0.45–0.20 eV, respectively. Generally, BEEF‐vdW gives the highest barrier and rev‐vdW‐DF2 gives the lowest barrier. The activation energies from all vdW approaches differ within 0.25 eV for Cu(111) and Ag(111) and 0.35 eV for Au(111) but the deviation for the reaction energies can reach 0.5 eV for Cu(111), 0.4 eV for Ag(111) and 0.25 eV for Au(111). It has been experimentally observed that at low surface coverages (<1 monolayer) iodobenzene dissociates at ∼175 K on Cu(111),^[81]^ ∼200 K on Ag(111)^[82]^ and 200–250 K on Au(111)^[83]^ by forming adsorbed phenyl group, The reactivity trend from our calculated PES is Cu(111)>Ag(111)>Au(111), which agrees well with the experimental observations. Similar with debromination, all methods give the correct trend between the three surfaces and the activation energy with respect to reaction energy also shows the BEP relation (see Figure 5 purple line). The experimental observations also imply that deiodination occurs faster than the desorption of iodobenzene on these three surfaces. This can be understood by the fact that the activation energy of deiodination is lower than the desorption energy of iodobenzene, which is correctly predicted by all functionals except BEEF‐vdW. Besides, the ignition temperatures for C−I bond scission in iodobenzene are significantly lower than C−Br bond scission, which are also indicated by the lower activation energies for deiodination (Figure 7) than debromination (Figure 6) on Cu(111), Ag(111) and Au(111), respectively.

It is clear that we need more experimental measurements of activation and reaction energies for a more robust evaluation of the precision of theoretical methods. As a final remark, on‐surface Ullmann coupling on Cu(111) and Ag(111) is often accompanied by the formation of organometallic structures incorporating native metal adatoms (originating from the substrate). Recently, it has been proposed that such adatoms may even participate in C−Br scission.^[84]^ This adds an additional layer of complexity to determining activation energies experimentally due to a non‐trivial dependence on reaction order. The scission of C−I bonds occurs at a lower temperature where adatoms are less prevalent. Therefore, it would be strategic to primarily focus experimental efforts on deiodination reactions to provide accurate estimations of activation energies.

## Conclusions

Five different vdW approaches have been employed for studying prototypical on‐surface synthesis reactions, namely dehydrogenation, debromination and deiodination. These methods were evaluated using using benzene, bromobenzene and iodobenzene on the (111) facets of the three coinage metals as model systems. Comparing the calculations to experimental values for the adsorption of benzene, all methods except BEEF‐vdW give accurate descriptions of adsorption heights on Ag(111) and Au(111), with only rev‐vdW‐DF2 closely resembling the adsorption on Cu(111). Overall, rev‐vdW‐DF2 yields the most accurate descriptions of adsorption heights and energies for benzene on the three surfaces, while BEEF‐vdW exhibits severe shortcomings. For the bromobenzene and iodobenzene adsorption on Ag(111) and Au(111), all functionals except BEEF‐vdW give roughly similar adsorption geometries. Assessing the reaction landscape for dehydrogenation on Cu(111), all functionals except BEEF‐vdW give reasonable activation energies compared with experimental estimations. For debromination and deiodination reactions, all of the five vdW approaches successfully describe the reactivity trend among the three surfaces, *i. e*., Cu(111)*>*Ag(111)*>*Au(111). Most notably, except for BEEF‐vdW, all methods match available experimental data for debromination on Cu(111), along with the anticipated hierarchy between halobenzene desorption and carbon‐halogen activation for both bromine and iodine. Additionally, BEEF‐vdW falls short to describe the deiodination reaction landscape on all these three surfaces. Additional experimental work is called for to provide a more rigorous evaluation of theoretical methods. Our work forms a foundation for future comparisons and serves as an initial guide for selecting density functionals for theoretical studies in the on‐surface synthesis field.

## Supporting Information Summary

The supporting information includes the calculated lattice constants *a* for bulk Cu, Ag and Au; the schematic representation of benzene adsorbed on Cu(111); the adsorption energies of benzene adsorbed on Cu(111), Ag(111) and Au(111) from different density functionals; the potential energy surface of benzene adsorbed on the three surfaces at hcp site as a function of benzene's height; the projected density of states of metal d, carbon, bromine and iodine p electrons in each adsorbed structure from different density functionals and the linear scaling relation of d‐band center shift to the height of benzene, bromobenzene and iodobenzene on three metal surfaces.

## Conflict of Interests

The authors declare no conflict of interest.

1

## Supporting information

As a service to our authors and readers, this journal provides supporting information supplied by the authors. Such materials are peer reviewed and may be re‐organized for online delivery, but are not copy‐edited or typeset. Technical support issues arising from supporting information (other than missing files) should be addressed to the authors.

Supporting Information

## Data Availability

All of the calculated structures can be found in Zenodo (https://doi.org/10.5281/zenodo.13865862).
