# Supplementary material for: A Density Functional Benchmark for Dehydrogenation and Dehalogenation Reactions on Coinage Metal Surfaces
Source: Chemphyschem. 2024 Nov 13;26(1):e202400865. doi: 10.1002/cphc.202400865 (PMC11747582; doi:10.1002/cphc.202400865)
Supplement: Supplementary file 1 — Supporting Information [file CPHC-26-e202400865-s001.pdf]

ChemPhysChem

Supporting Information

A Density Functional Benchmark for Dehydrogenation and Dehalogenation Reactions on Coinage Metal Surfaces

Lin Chen, Johanna Rosen, and Jonas Björk*

Supporting Information: A density functional benchmark for dehydrogenation and dehalogenation reactions on coinage metal surfaces

Lin Chen,[1] Johanna Rosen,[1] Jonas Björk[1*]

[1] *Materials Design Division, Department of Physics, Chemistry and Biology, IFM, Linköping University, 58183, Linköping, Sweden*

E-mail: jonas.bjork@liu.se

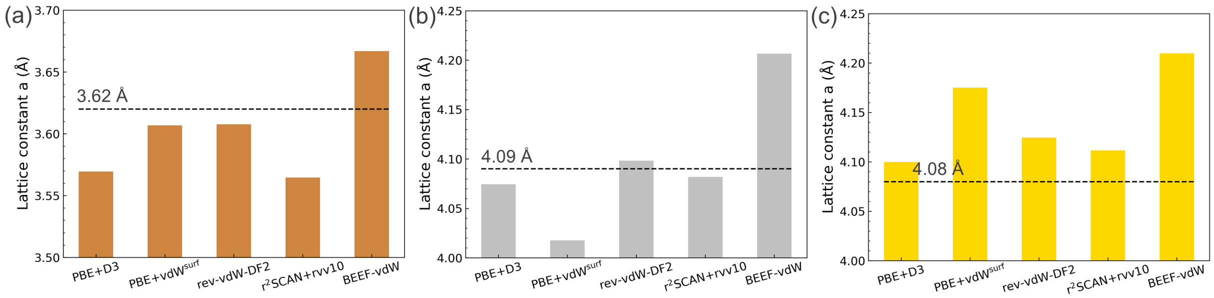

Figure S1: The calculated lattice constant a for bulk (a) Cu, (b) Ag and (c) Au from different density functionals. The horizontal line indicates the experimental value.

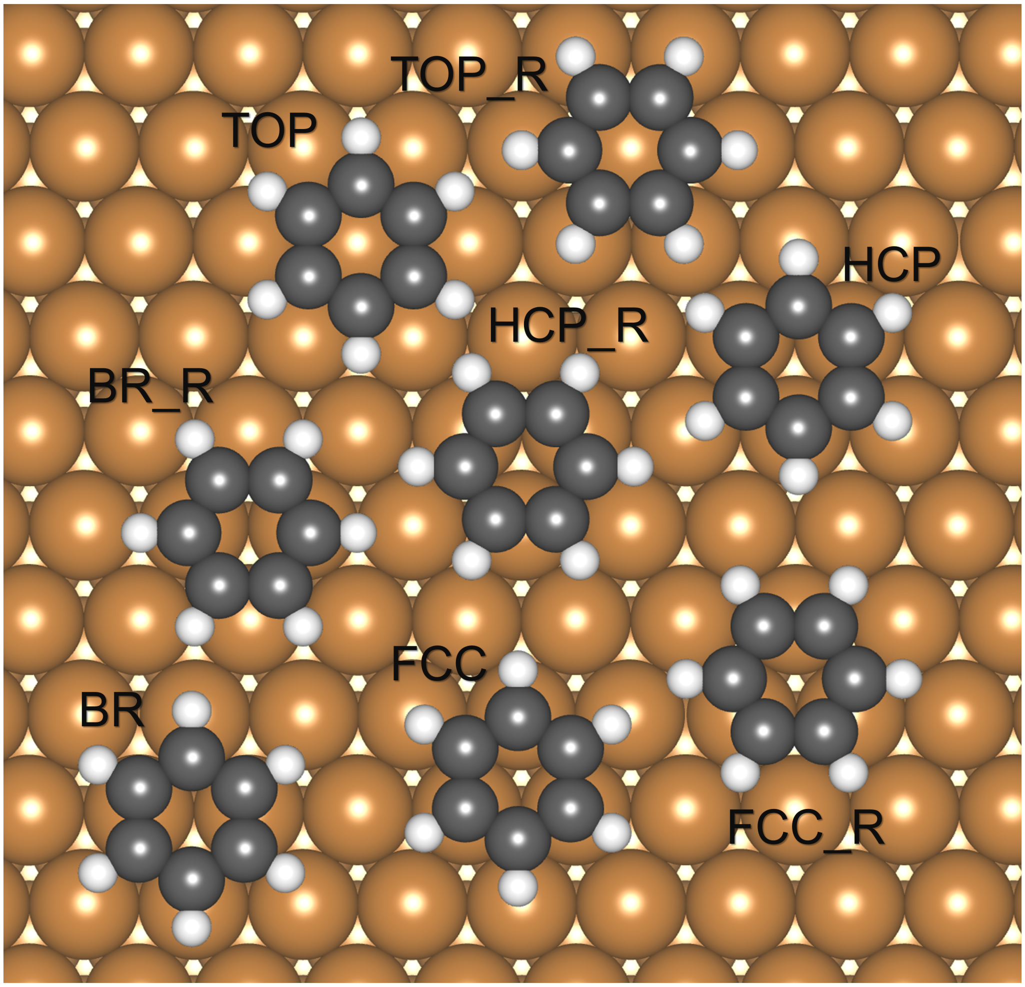

Figure S2: Schematic representation of benzene adsorbed on the high symmetry sites of Cu(111). Two angular orientations (0 and 30 degrees with respect to <$1\bar{1}0$> direction) for each site are considered in this work. BR represents the bridge site. The notation with (or without) R indicates the 30 (or 0)-degree rotation. Atom color codes: Cu (peru), C (dimgray) and H(white).

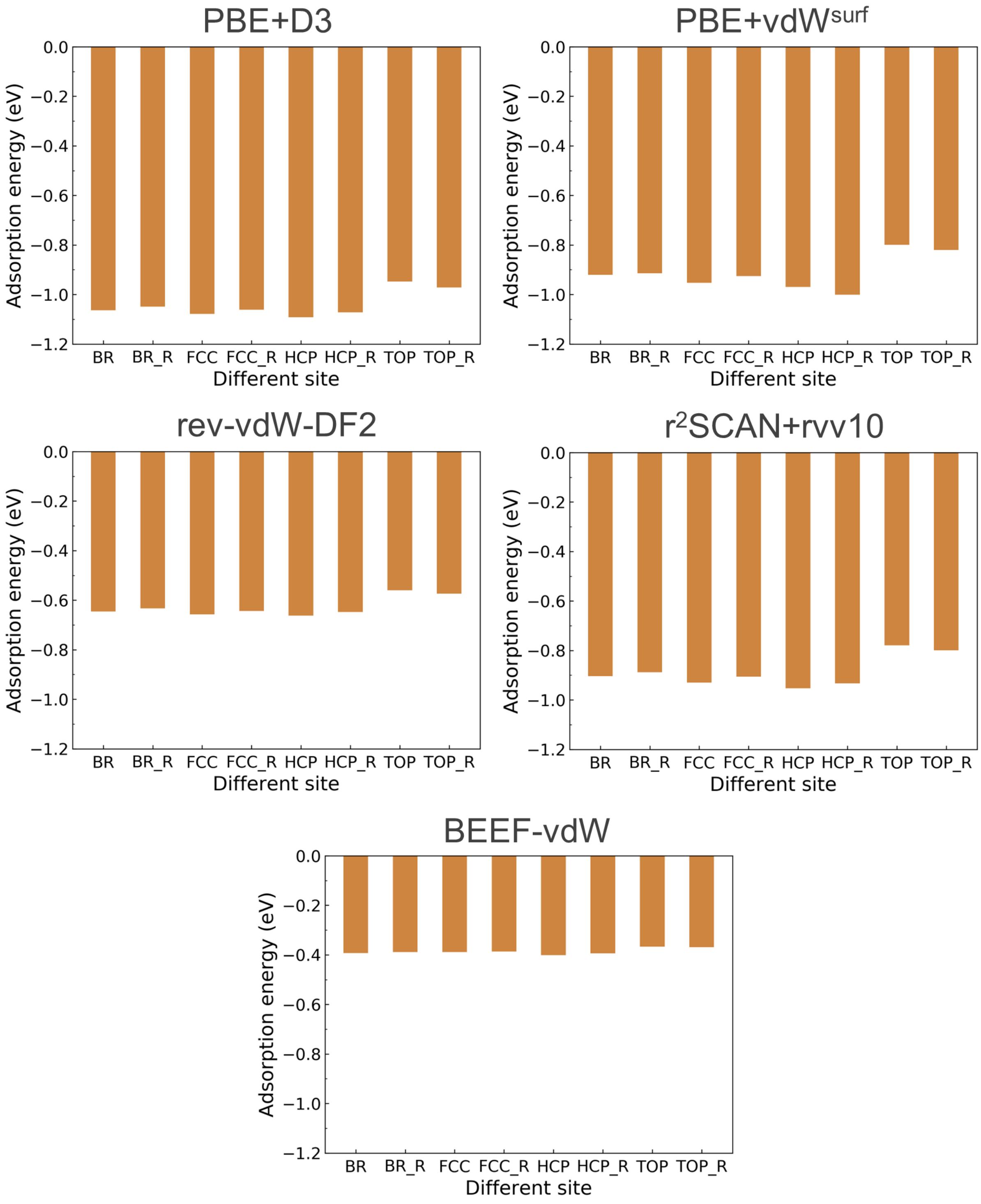

Figure S3: The adsorption energy of benzene on the high symmetry sites of Cu(111) from different density functionals.

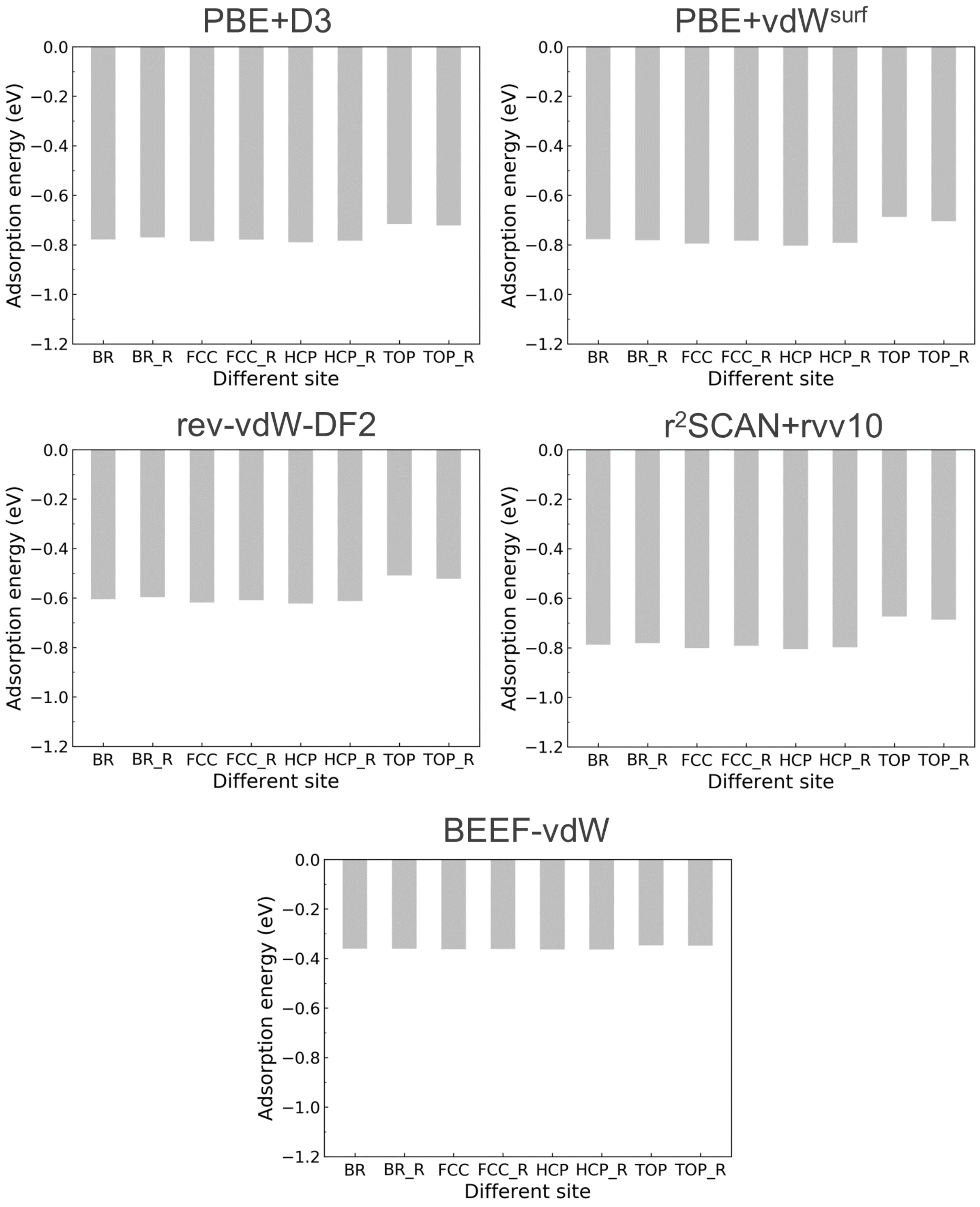

Figure S4: The adsorption energy of benzene on the high symmetry sites of Ag(111) from different density functionals.

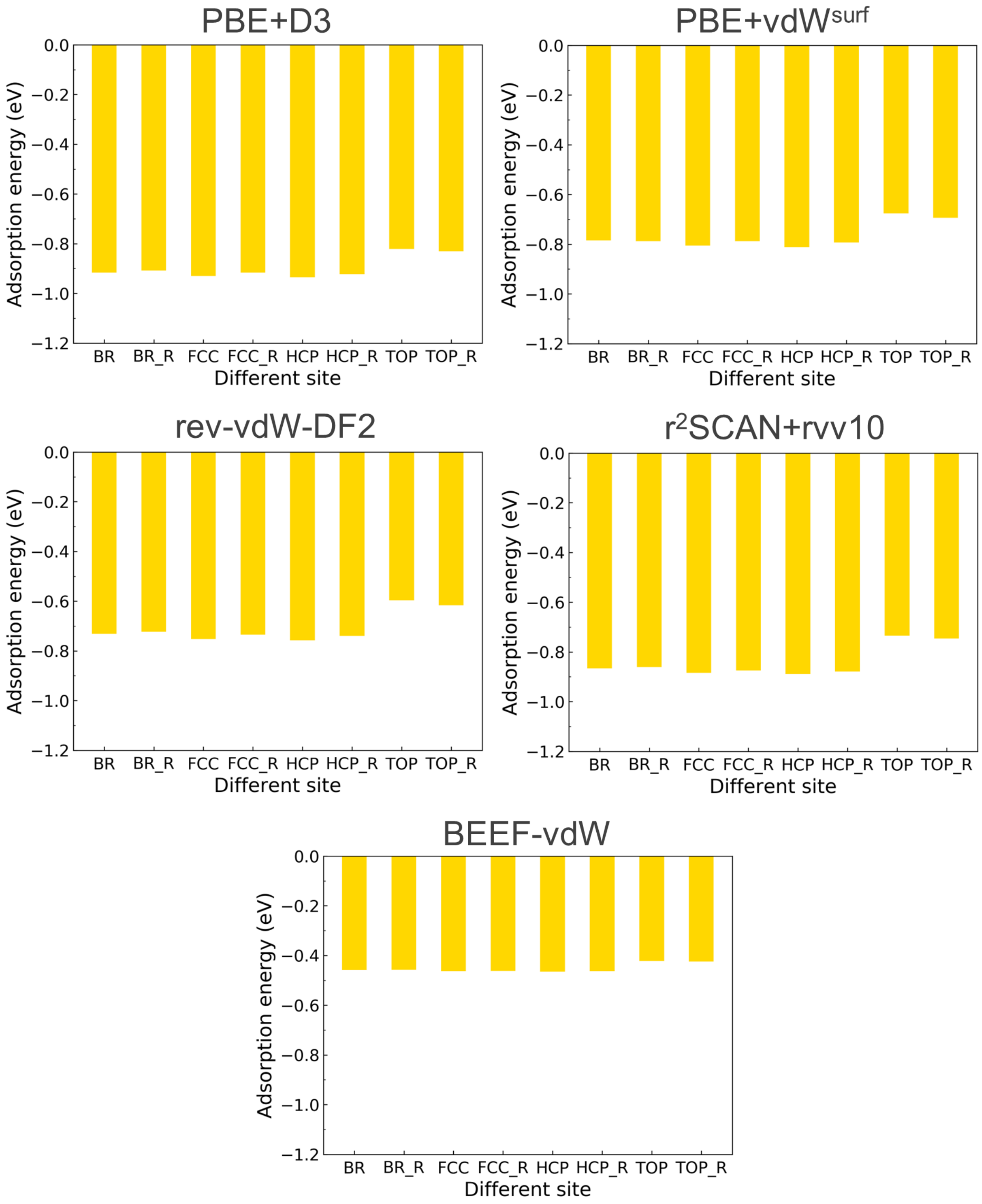

Figure S5: The adsorption energy of benzene on the high symmetry sites of Au(111) from different density functionals.

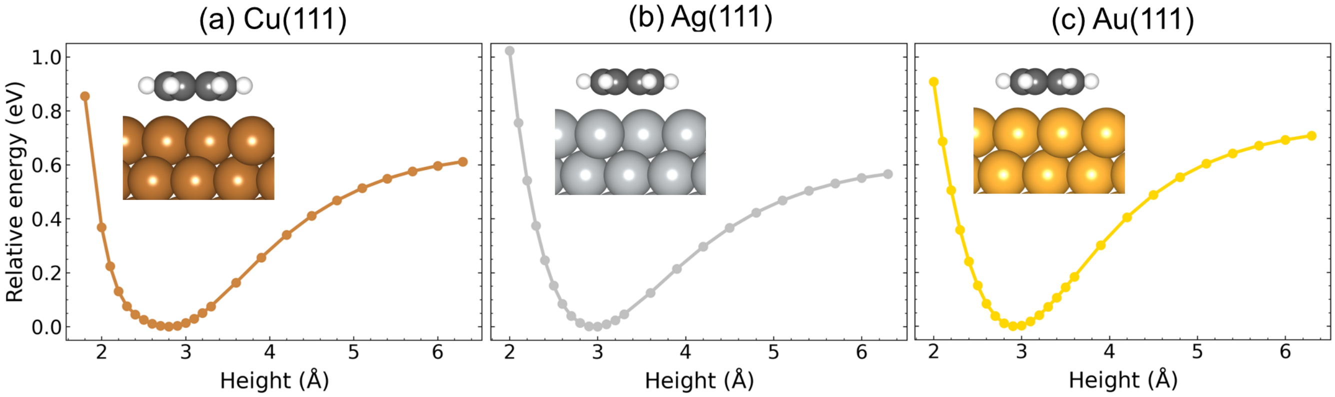

Figure S6: The potential energy surface of benzene adsorbed on a) Cu(111), (b) Ag(111) and (c) Au(111) at hcp site as a function of the benzene's height from rev-vdW-DF2. The height of benzene is determined by calculating the averaged carbon-metal(top layer metal atoms) distance. Constrained relaxations at each height are performed by fixing benzene's center of mass in z direction. The structure with the lowest energy is further full-relaxed and the relaxed structure is shown as the inset. The lines are shown as a guide to the eye. The figures share the same range of y axis. Atom color codes: Au (gold), Ag (silver), Cu (peru), C (dimgray) and H(white).

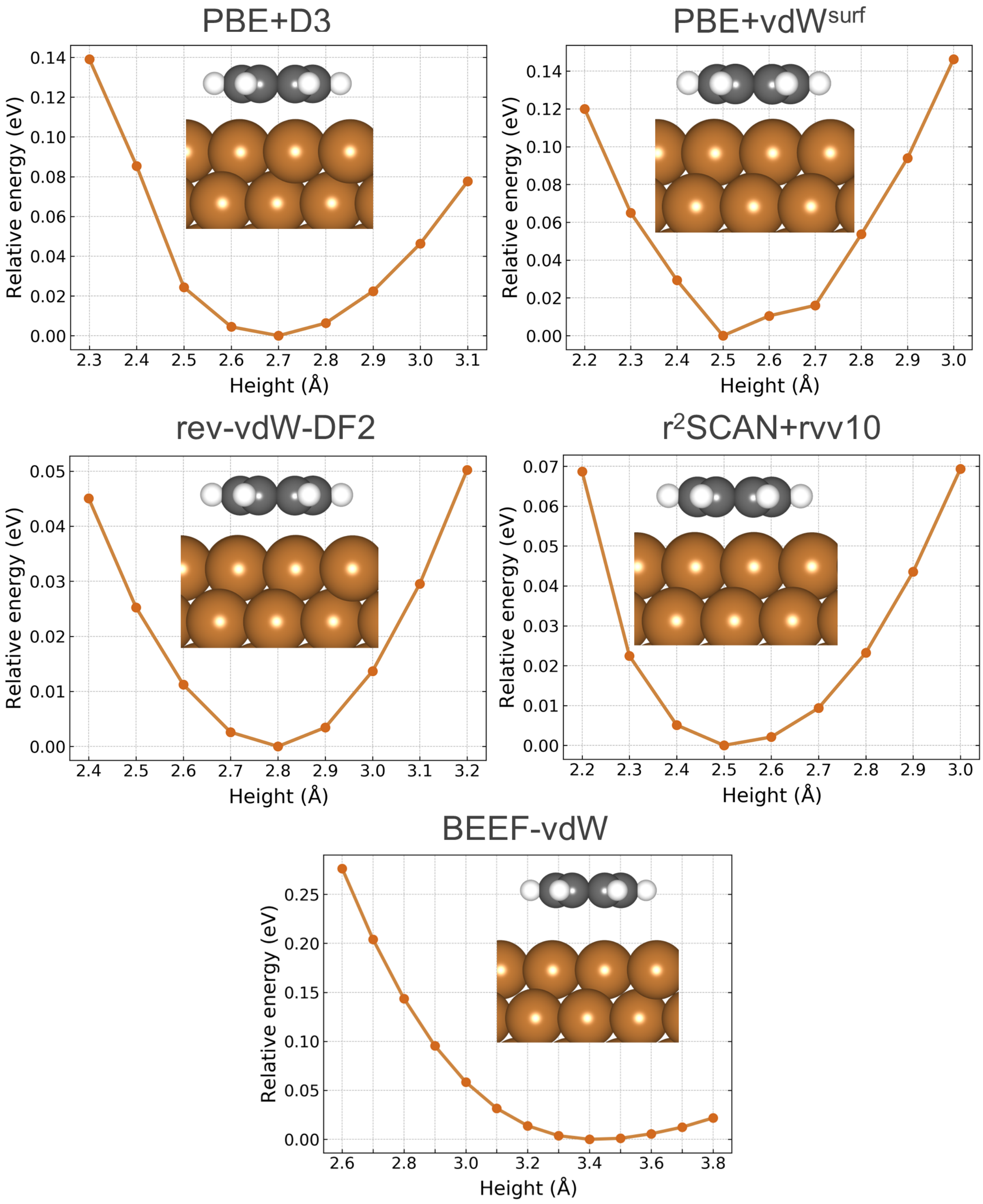

Figure S7: The potential energy surface of benzene adsorbed on Cu(111) at hcp site as a function of the benzene height from different density functionals. The height of benzene is determined by calculating the averaged carbon-metal(top layer metal atoms) distance. A constrained relaxation at each height is performed by fixing benzene's center of mass in z direction. The structure with the lowest energy is further full-relaxed and the relaxed structure is shown as the inset. The lines are shown as a guide to the eye. Atom color codes: Cu (peru), C (dimgray) and H(white).

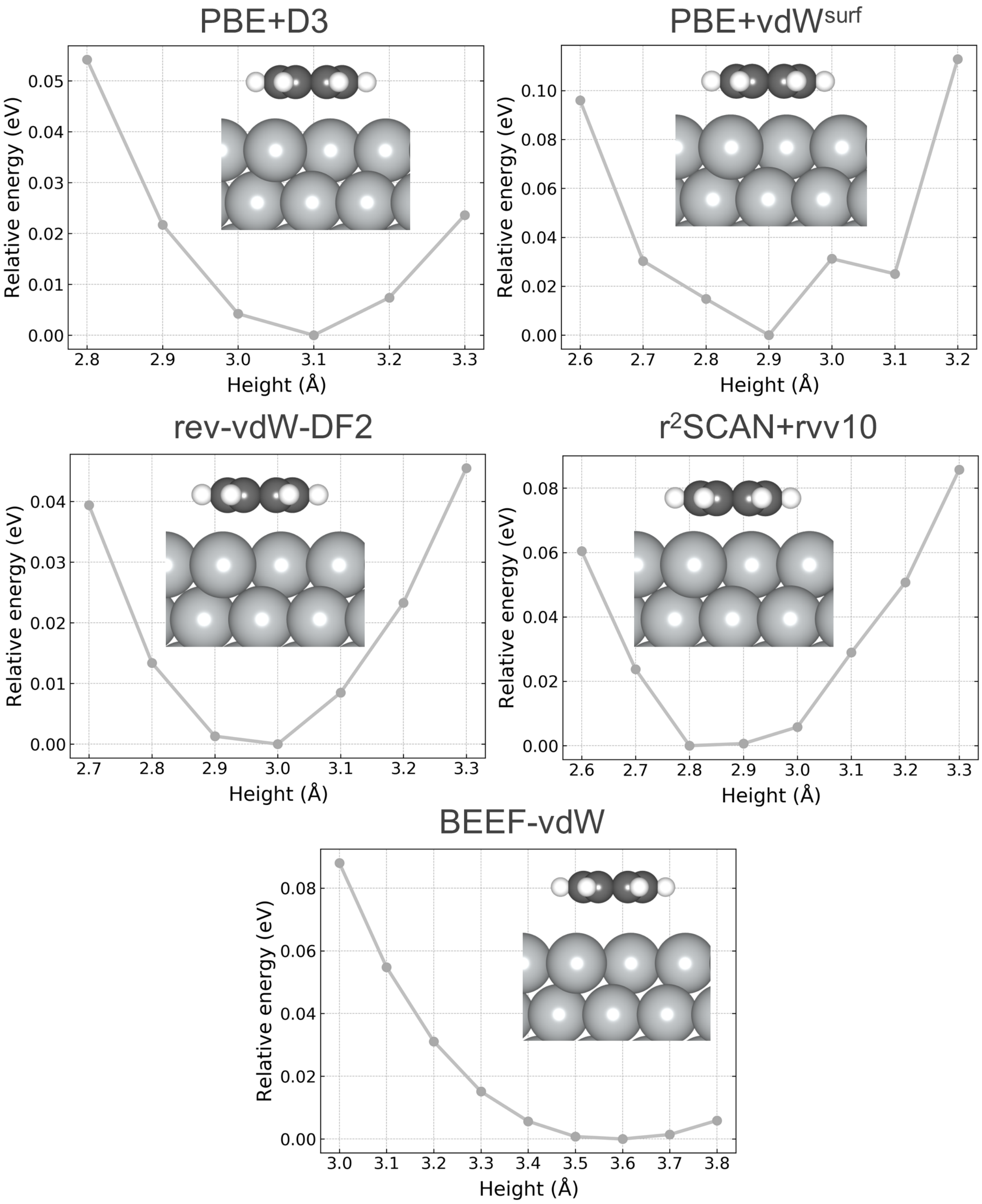

Figure S8: The potential energy surface of benzene adsorbed on Ag(111) at hcp site as a function of the benzene height from different density functionals. The height of benzene is determined by calculating the averaged carbon-metal(top layer metal atoms) distance. A constrained relaxation at each height is performed by fixing benzene's center of mass in z direction. The structure with the lowest energy is further full-relaxed and the relaxed structure is shown as the inset. The lines are shown as a guide to the eye. Atom color codes: Ag (silver), C (dimgray) and H(white).

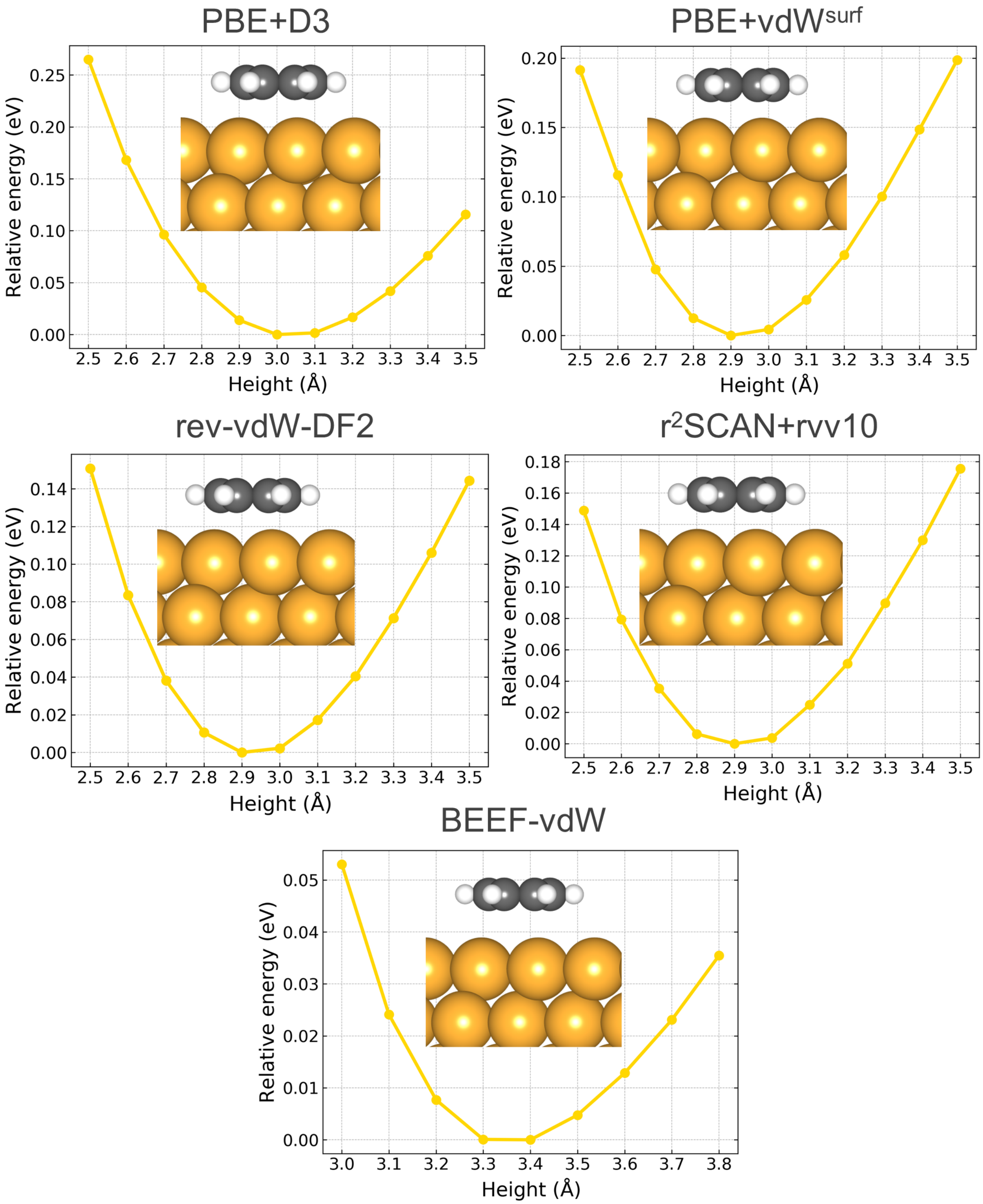

Figure S9: The potential energy surface of benzene adsorbed on Au(111) at hcp site as a function of the benzene height from different density functionals. The height of benzene is determined by calculating the averaged carbon-metal(top layer metal atoms) distance. A constrained relaxation at each height is performed by fixing benzene's center of mass in z direction. The structure with the lowest energy is further full-relaxed and the relaxed structure is shown as the inset. The lines are shown as a guide to the eye. Atom color codes: Au (gold), C (dimgray) and H(white).

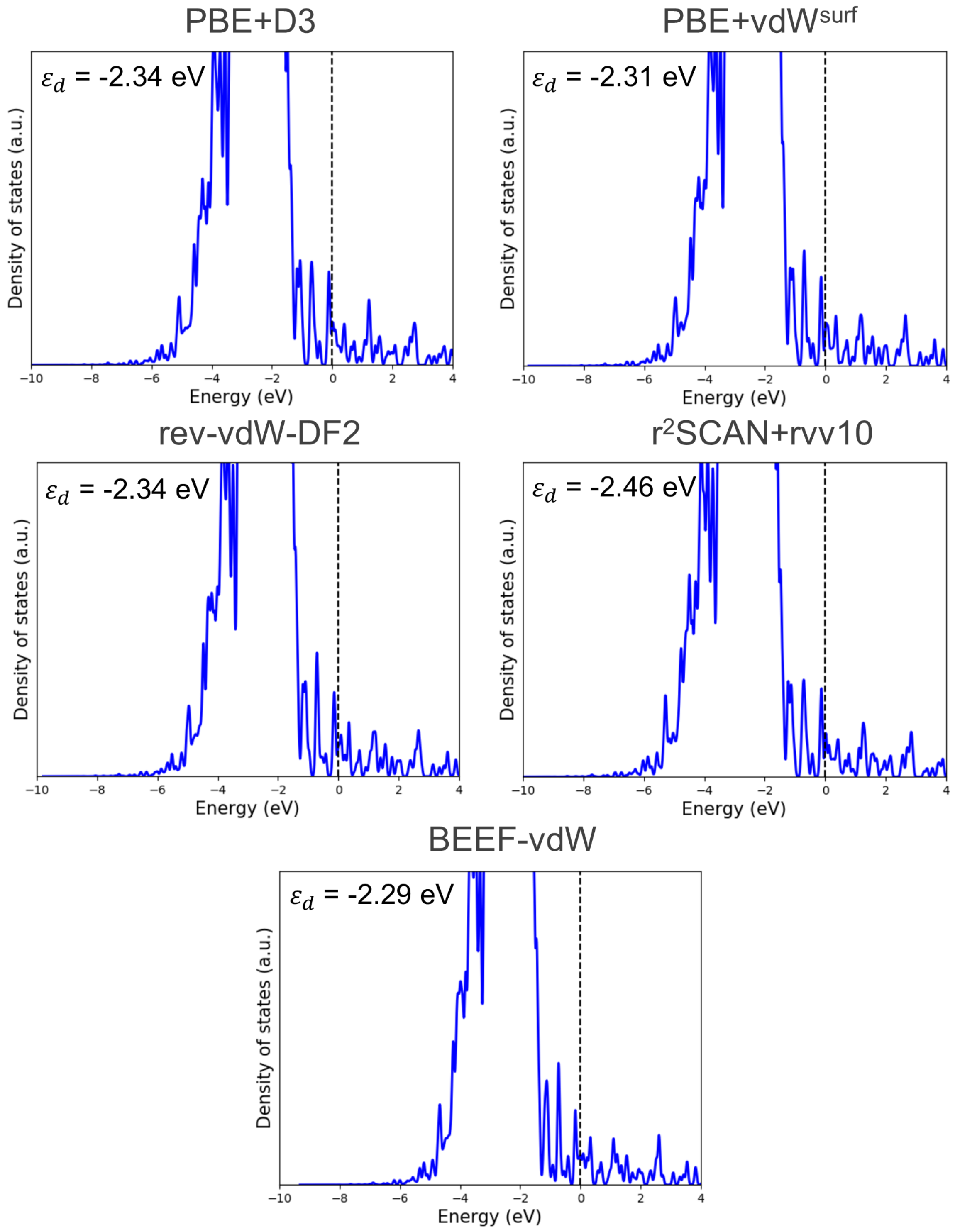

Figure S10: Density of states of Cu 3d electrons from different functionals. The dashed line denotes the Fermi-energy level. The d-band center (ε_d) is shown in each figure.

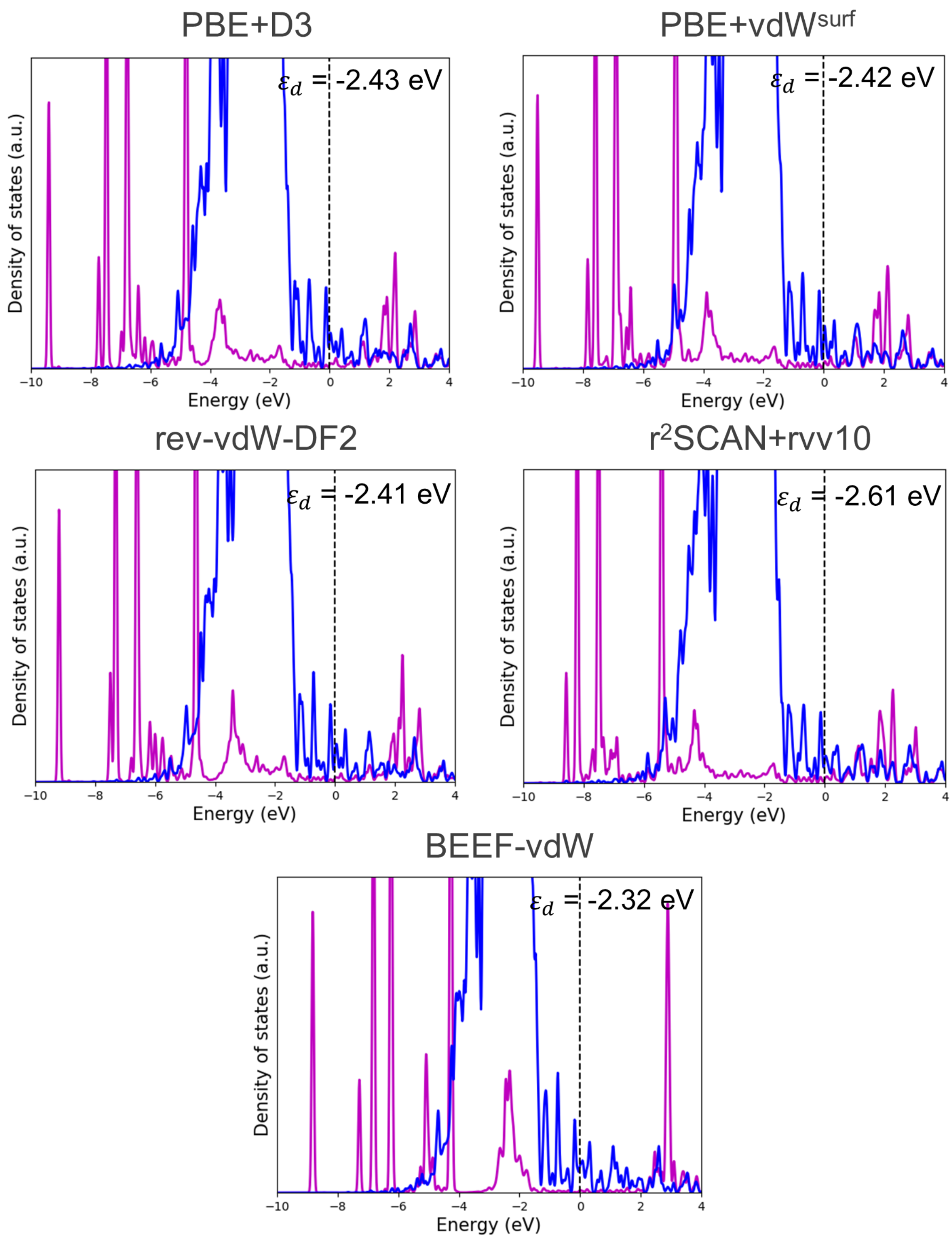

Figure S11: Density of states of Cu 3d (blue) and C 2p (purple) electrons of Benzene adsorbed on Cu(111) from different functionals. The dashed line denotes the Fermi-energy level. The d-band center (ε_d) is shown in each figure.

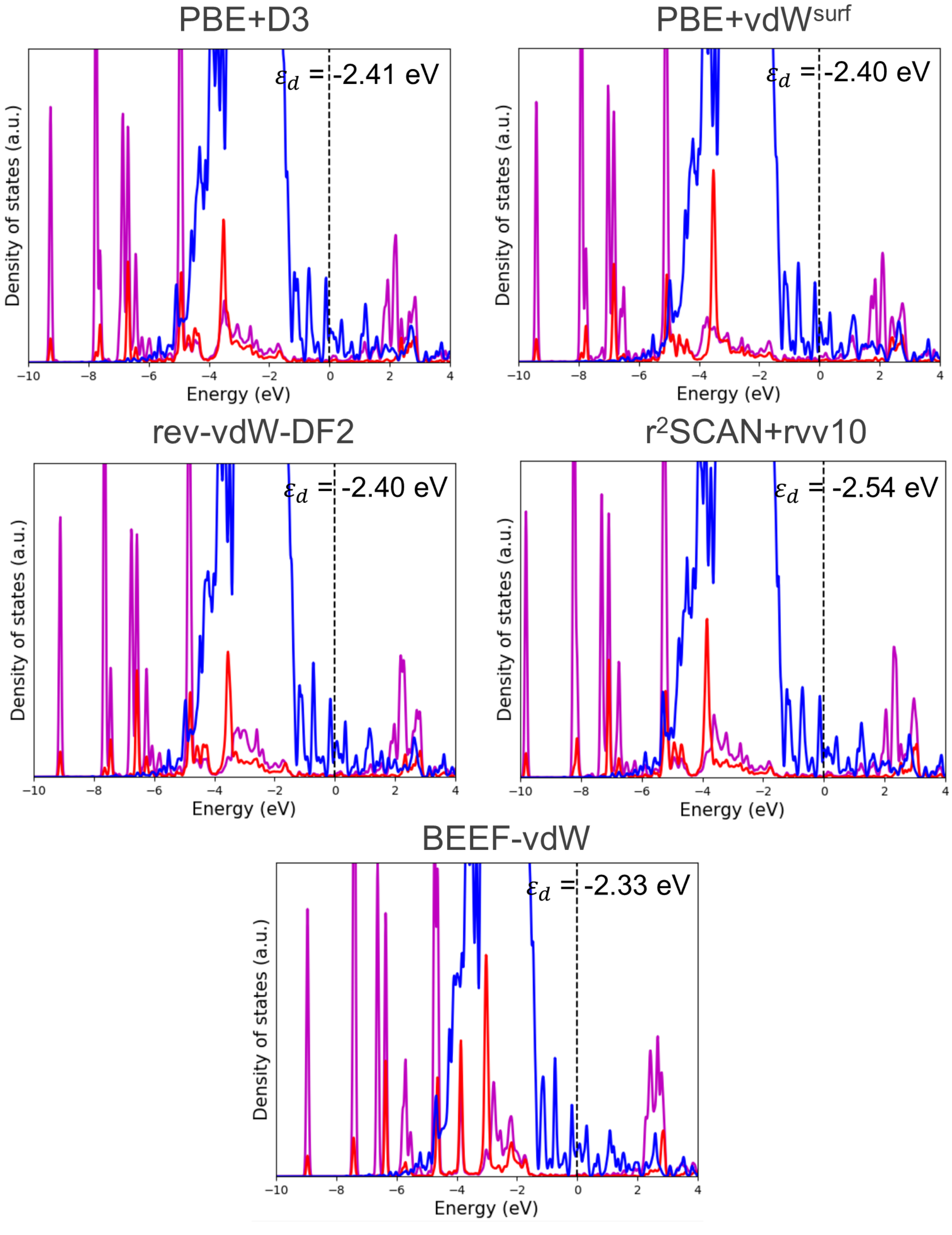

Figure S12: Density of states of Cu 3d (blue), C 2p (purple) and Br 4p (red) electrons of bromobenzene adsorbed on Cu(111) from different functionals. The dashed line denotes the Fermi-energy level. The d-band center (ε_d) is shown in each figure.

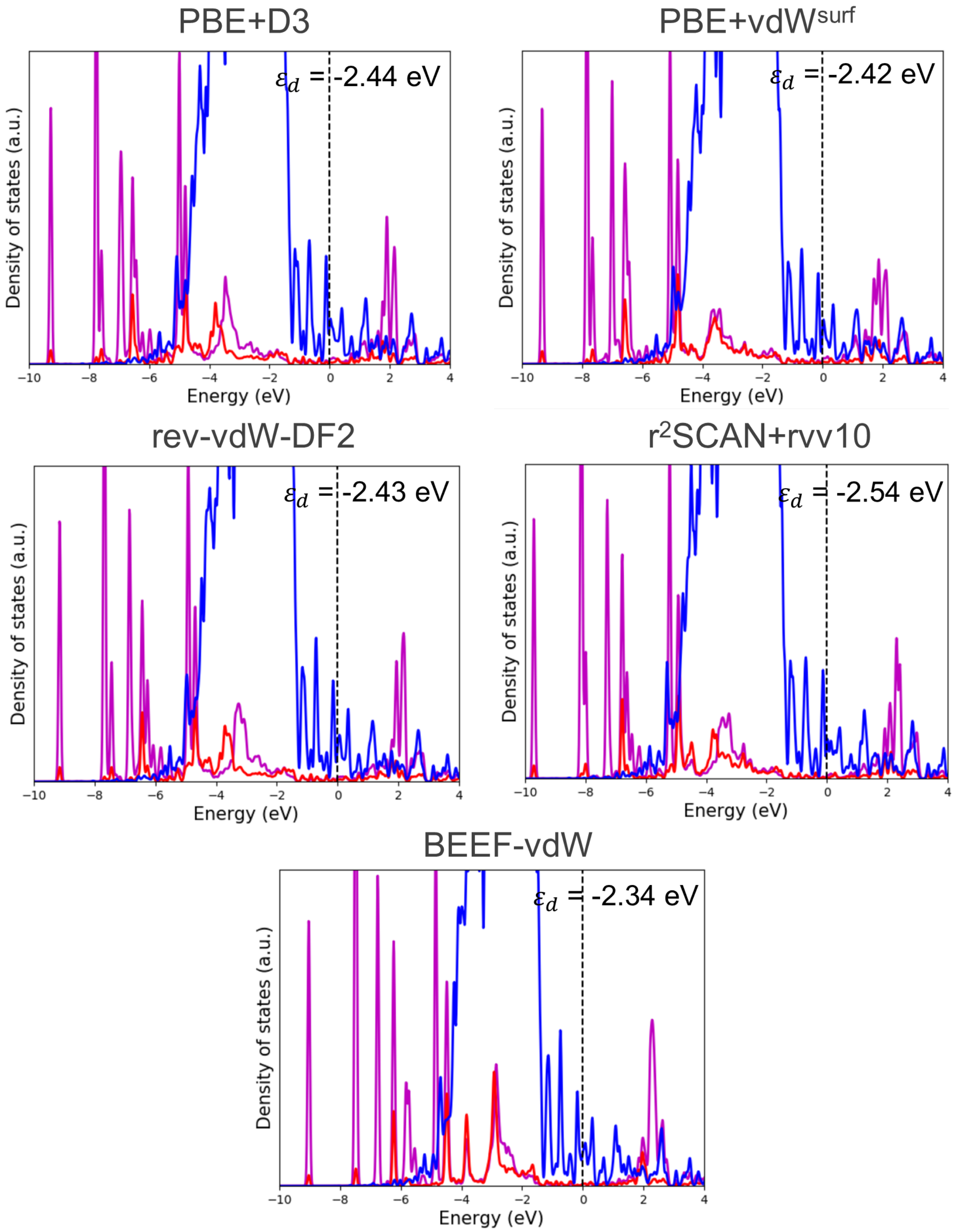

Figure S13: Density of states of Cu 3d (blue), C 2p (purple) and I 5p (red) electrons of iodobenzene adsorbed on Cu(111) from different functionals. The dashed line denotes the Fermi-energy level. The d-band center (ε_d) is shown in each figure.

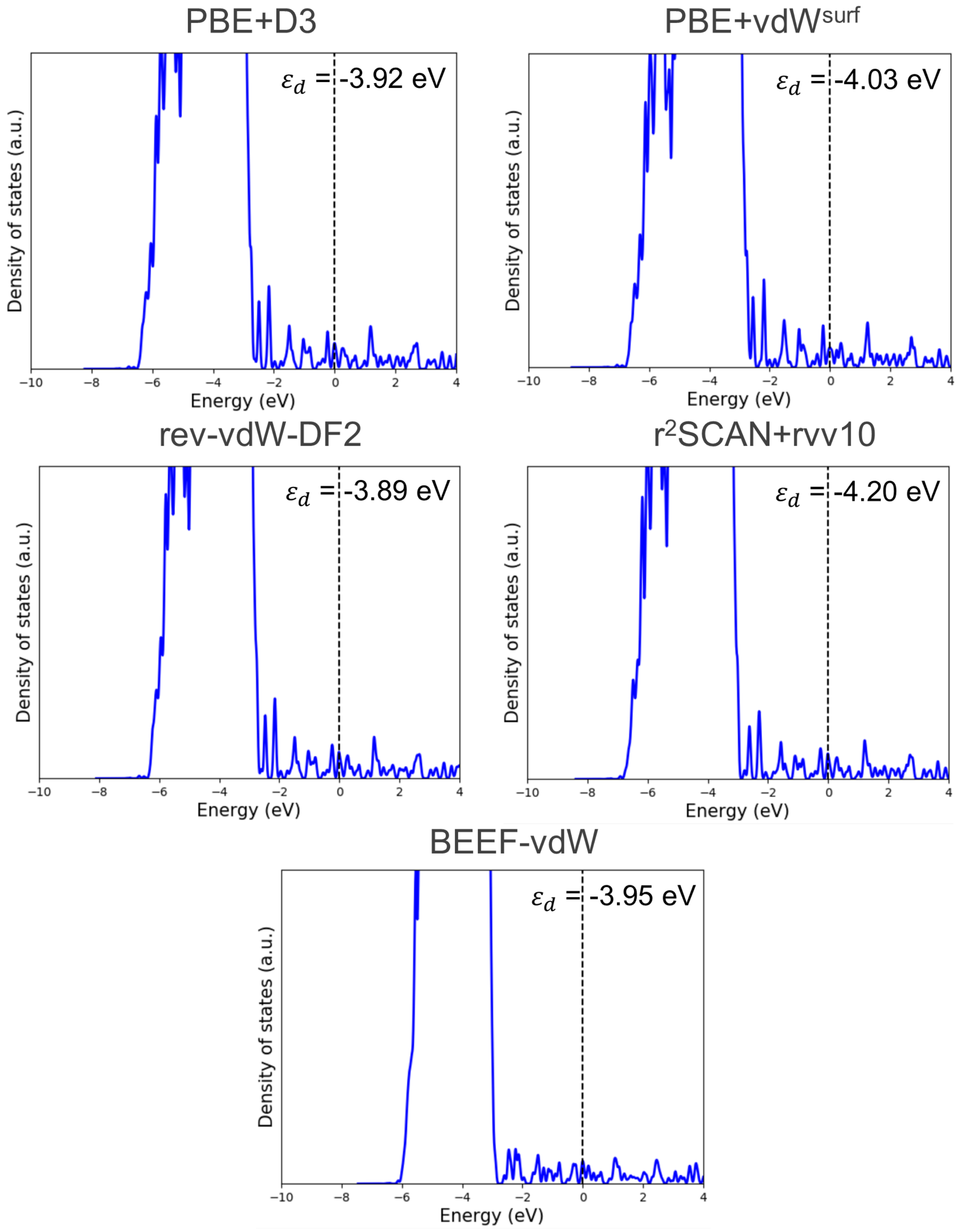

Figure S14: Density of states of Ag 4d electrons from different functionals. The dashed line denotes the Fermi-energy level. The d-band center (ε_d) is shown in each figure.

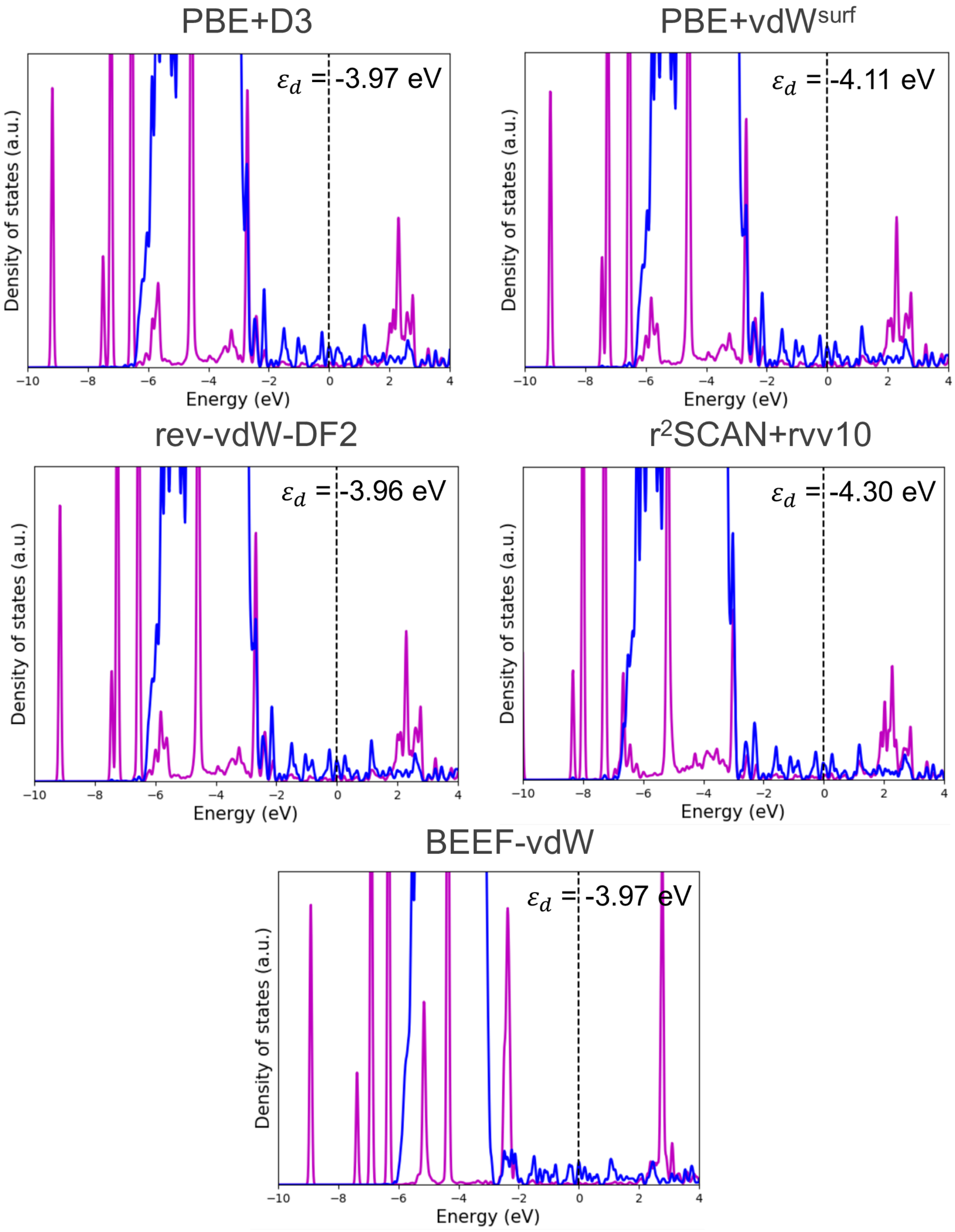

Figure S15: Density of states of Ag 4d (blue) and C 2p (purple) electrons of Benzene adsorbed on Ag(111) from different functionals. The dashed line denotes the Fermi-energy level. The d-band center (ε_d) is shown in each figure.

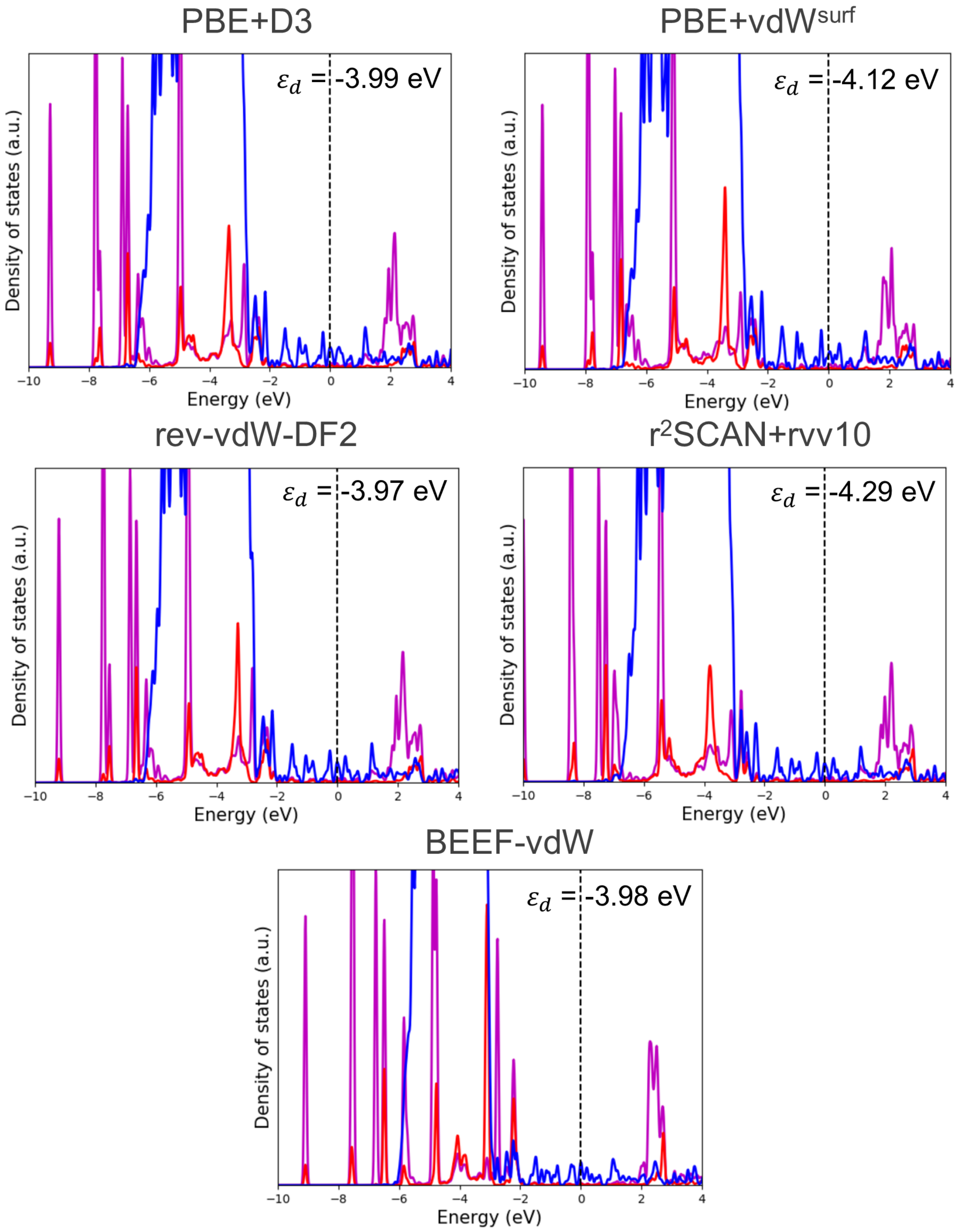

Figure S16: Density of states of Ag 4d (blue), C 2p (purple) and Br 4p (red) electrons of bromobenzene adsorbed on Ag(111) from different functionals. The dashed line denotes the Fermi-energy level. The d-band center (ε_d) is shown in each figure.

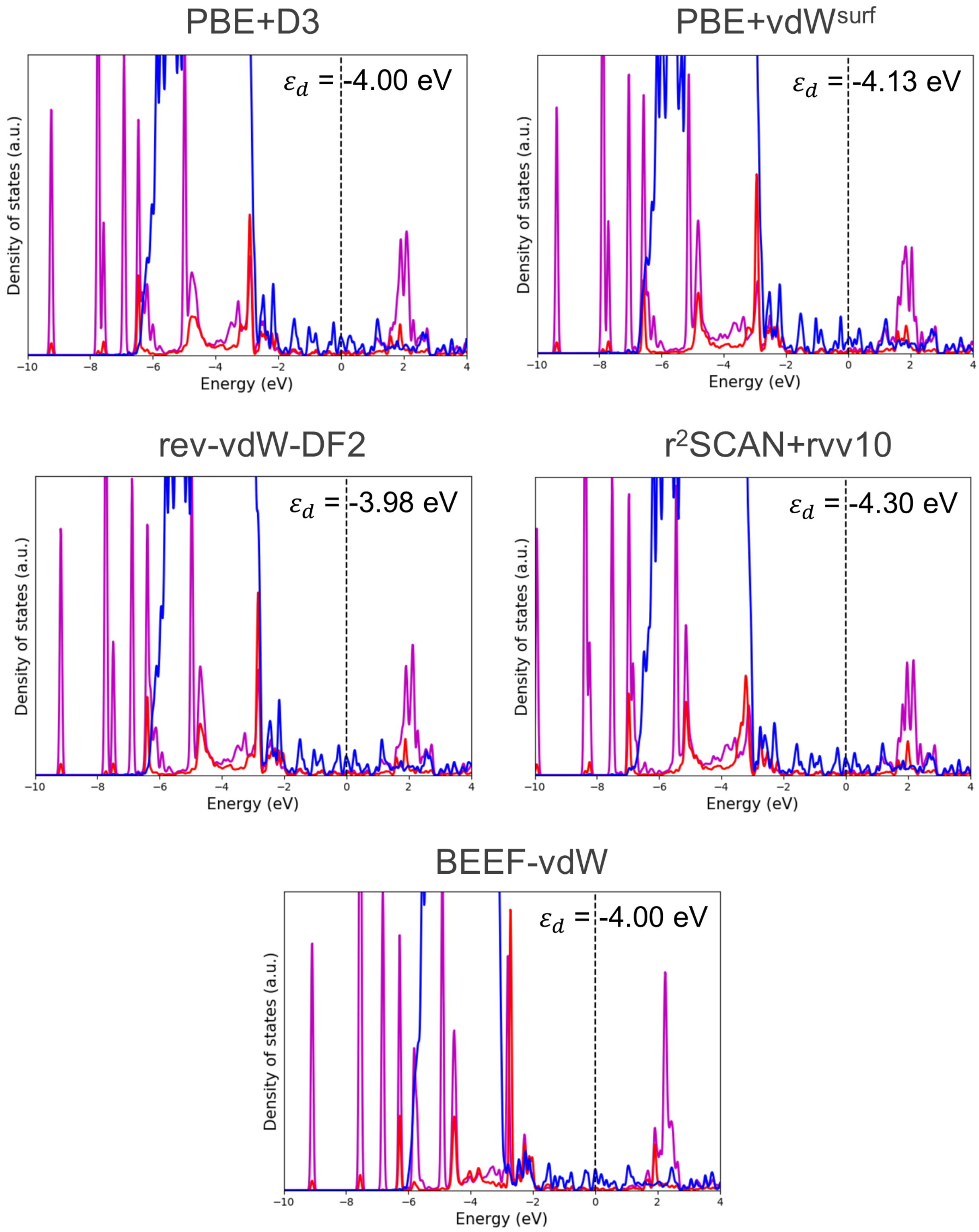

Figure S17: Density of states of Ag 4d (blue), C 2p (purple) and I 5p (red) electrons of iodobenzene adsorbed on Ag(111) from different functionals. The dashed line denotes the Fermi-energy level. The d-band center (ε_d) is shown in each figure.

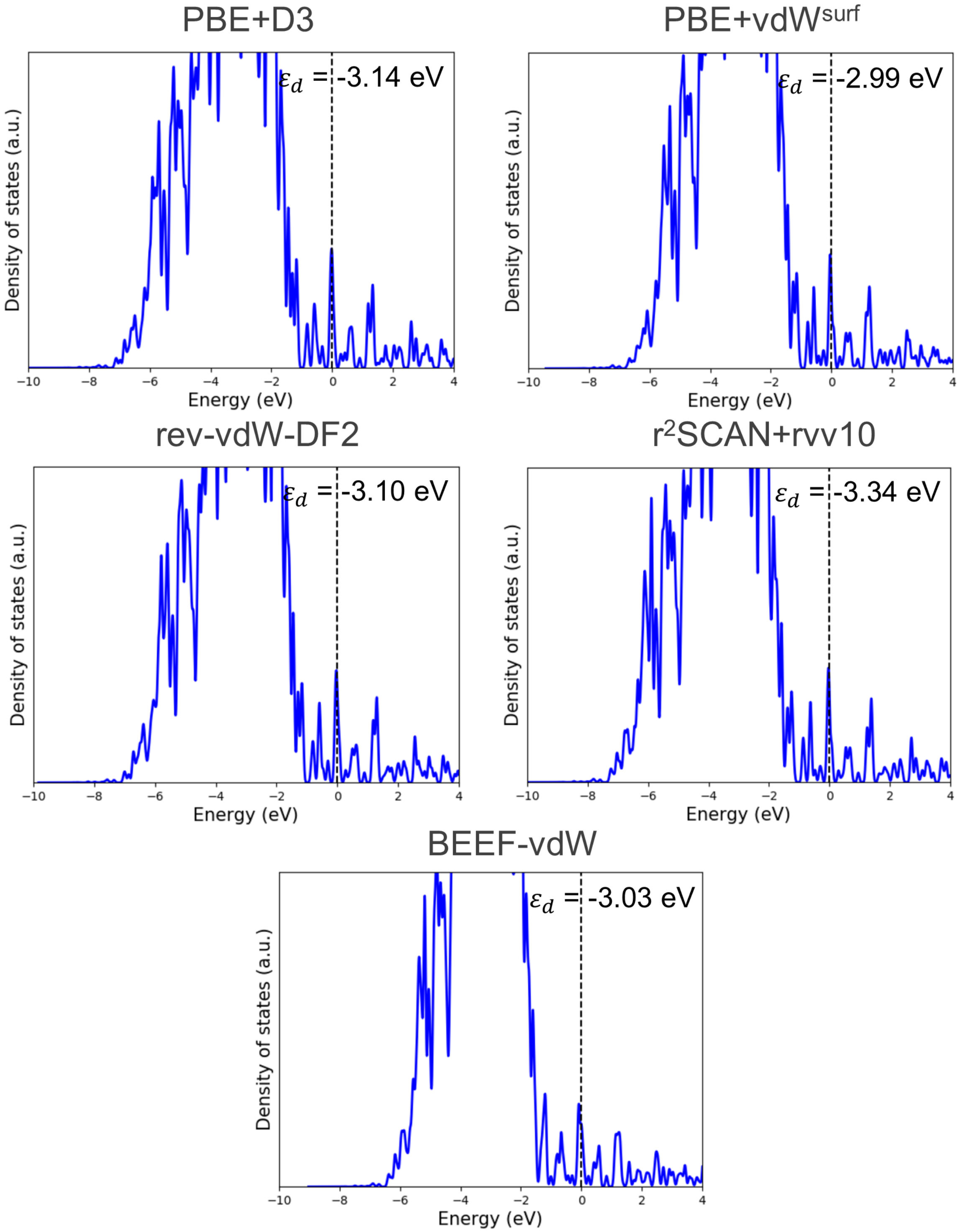

Figure S18: Density of states of Au 5d electrons from different functionals. The dashed line denotes the Fermi-energy level. The d-band center (ε_d) is shown in each figure.

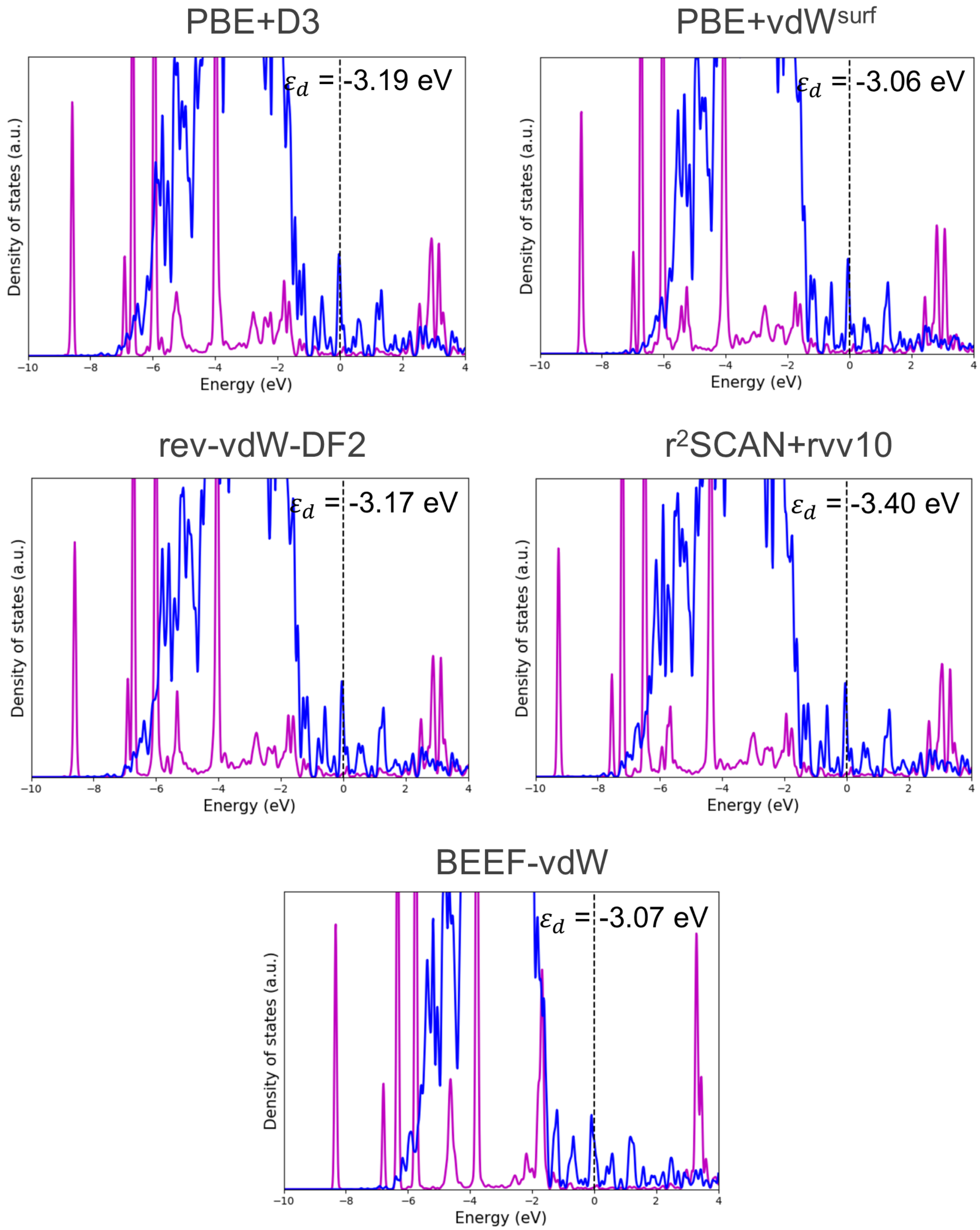

Figure S19: Density of states of Au 5d (blue) and C 2p (purple) electrons of Benzene adsorbed on Au(111) from different functionals. The dashed line denotes the Fermi-energy level. The d-band center (ε_d) is shown in each figure.

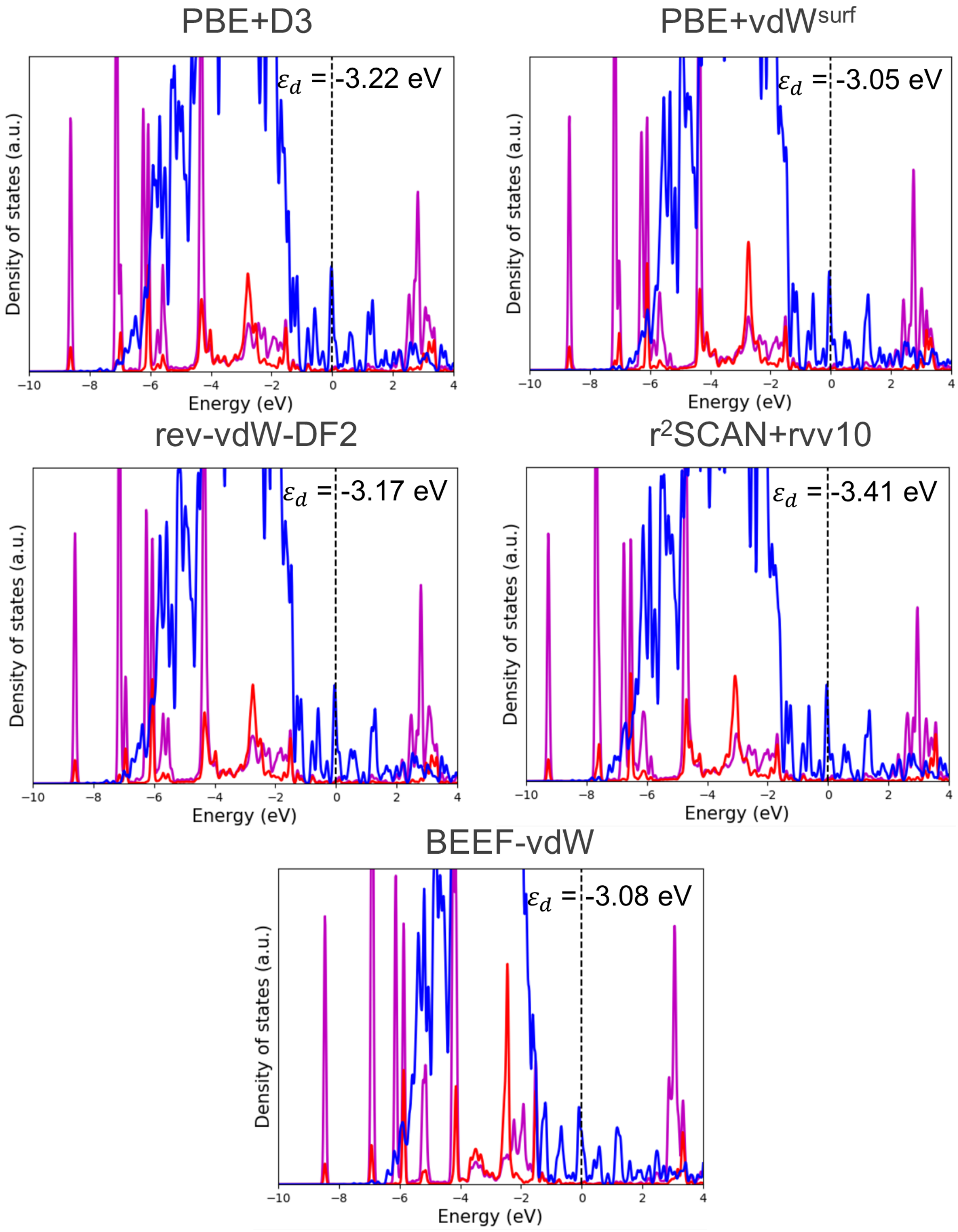

Figure S20: Density of states of Au 5d (blue), C 2p (purple) and Br 4p (red) electrons of bromobenzene adsorbed on Au(111) from different functionals. The dashed line denotes the Fermi-energy level. The d-band center (ε_d) is shown in each figure.

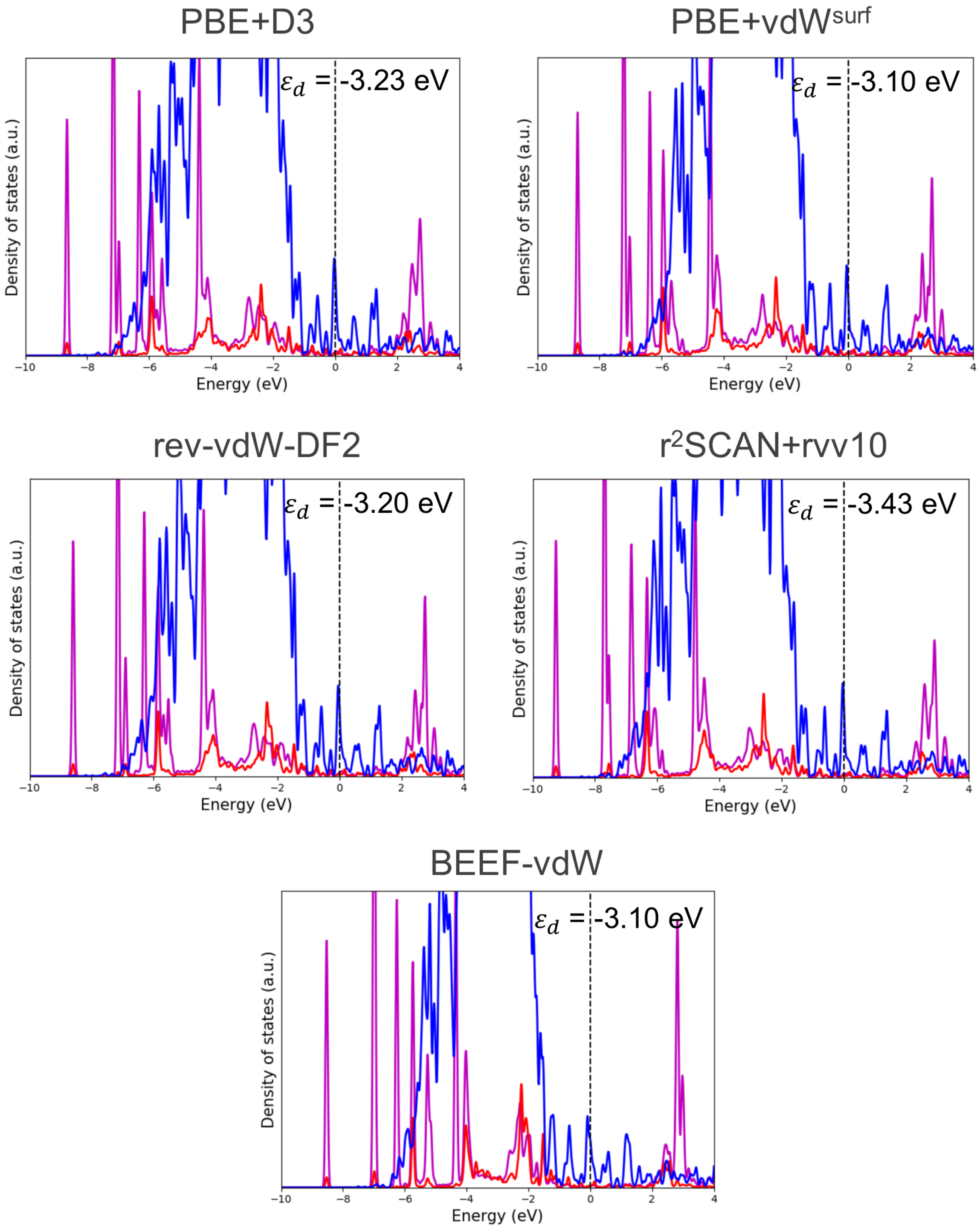

Figure S21: Density of states of Au 5d (blue), C 2p (purple) and I 5p (red) electrons of iodobenzene adsorbed on Au(111) from different functionals. The dashed line denotes the Fermi-energy level. The d-band center (ε_d) is shown in each figure.

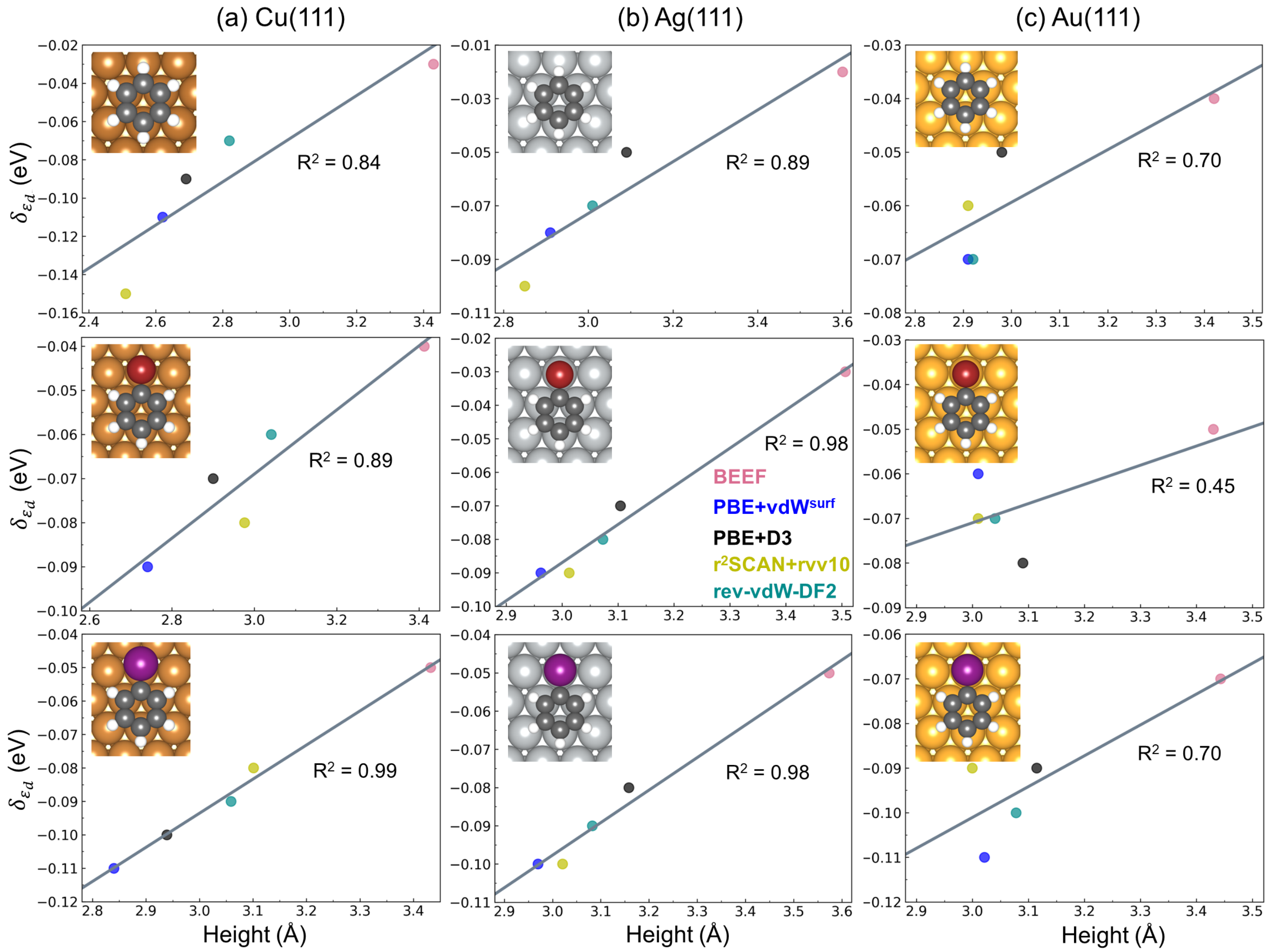

Figure S22: Linear scaling relationship of the shifting of the d-band center with respect to the height of benzene, bromobenzene and iodobenzene on (a) Cu(111), b) Ag(111) and (c) Au(111), respectively. The average height of carbon atoms is considered as the height for all of the three molecules. The configurations from rev-vdW-DF2 are shown in the inset. The marker color codes: PBE+D3 (black), PBE+vdW^{surf} (blue), rev-vdW-DF2 (green), r^{2}SCAN+rVV10 (yellow) and BEEF-vdW (pink). Atom color codes: Au (gold), I (purple), Ag (silver), Br (brown), Cu (peru), C (dimgray) and H(white).
